# Comparative Genomic Analysis of Livestock-Derived *Campylobacter jejuni*: Antimicrobial Resistance, Virulence, Mobile Genetic Elements, and Genetic Relatedness

**DOI:** 10.4014/jmb.2411.11044

**Published:** 2025-02-14

**Authors:** Jae-Uk An, Junbum Lee, Seongbeom Cho, Hyokeun Song

**Affiliations:** College of Veterinary Medicine and Research Institute for Veterinary Science, Seoul National University, Seoul 08826, Republic of Korea

**Keywords:** Campylobacter, whole genome sequencing (WGS), antimicrobial resistance (AMR), virulence factor (VF), mobile genetic element (MGE), core genome multi-locus sequence typing (cgMLST)

## Abstract

*Campylobacter jejuni* is a major cause of foodborne illnesses, and its increasing antimicrobial resistance (AMR) poses serious public health risks. Owing to their high genetic diversity and frequent intraspecific recombination, understanding the virulence traits of *Campylobacter* remains challenging. We elucidated the resistance and virulence mechanisms of *C. jejuni* in livestock using comparative genomic and phenotypic analyses. We analyzed *C. jejuni* strains isolated from chicken meat, chicken slaughterhouses, and dairy cattle farms. High resistance rates were observed for nalidixic acid, ciprofloxacin, and tetracycline. The chicken-derived strains showed significantly higher tetracycline resistance and marginally higher nalidixic acid resistance, whereas the cattle-derived strains showed marginally higher ciprofloxacin resistance. The key AMR determinants included *gyrA* and *tet(O)*, which were correlated with resistance phenotypes. Ten virulence factor families were identified with prevalences exceeding 90%. Biofilm formation was observed in 31.9% of strains and correlated with flagella-associated virulence factors. Eighteen plasmid types were detected, primarily in the pTet family, which carried various AMR genes and components of the Type IV secretion system, potentially facilitating the co-transfer of resistance and virulence traits. Conjugation experiments confirmed the horizontal transfer of two pTet plasmid types into the wild-type *C. jejuni* strain. Further, our analyses revealed over 95% genetic similarity with European *C. jejuni* strains in a public database—supporting the hypothesis of zoonotic transmission via global food chains—and the zoonotic risks of livestock-derived *Campylobacter jejuni*. These findings emphasize the need for extended global surveillance to mitigate the risk of zoonotic transmission.

## Introduction

*Campylobacter* spp. are widely recognized as the primary cause of foodborne bacterial diarrheal diseases [1]. Human transmission mainly occurs through meat consumption following slaughter and food processing, with approximately 79% of Campylobacteriosiscases in humans reported to be foodborne [2, 3]. *Campylobacter* is highly infectious and infections can occur with relatively low exposure, as few as 500–800 colony-forming units (CFU) [4]. According to FoodNet surveillance data, the U.S. Food and Drug Administration (FDA) reports an estimated 20 cases of campylobacteriosis per 100,000 people annually (FoodNet Fast: wwwn.cdc.gov/FoodNetFast). The use of antibiotics is generally unnecessary for treating uncomplicated cases of campylobacteriosis but is essential for managing severe, systemic, or chronic infections [5]. Macrolides are the preferred drugs for the treatment of intestinal *Campylobacter* infections. Fluoroquinolones are often selected for the empirical treatment of adults with suspected bacterial gastroenteritis, whereas tetracyclines serve as second-line treatment for intestinal *Campylobacter* infections [6]. In cases of severe *Campylobacter* bacteremia and other systemic infections, intravenous aminoglycosides and carbapenems are considered viable treatment options [7]. The prevalence of antimicrobial-resistant *Campylobacter* has increased, making it a growing public health concern worldwide. In particular, the rate of quinolone-resistant *Campylobacter* is continually increasing, although it varies between countries, and in many countries, fluoroquinolone-resistant *Campylobacter* is a concern [6]. The World Health Organization (WHO) has reported 12 pathogens as needing new antimicrobials research due to rising resistance, with fluoroquinolone-resistant *Campylobacter* designated as a pathogen of “High” priority and significant concern [8].

The virulence factors possessed by *Campylobacter* include cellular motility, adhesion, host cell invasion, and toxin formation, all of which play important roles in the pathogenesis of *Campylobacter* [9]. Adhesion of *Campylobacter* to the host intestinal epithelium is essential for colonization and can subsequently facilitate interactions between bacterial and eukaryotic host cells, leading to invasion [10]. Among the virulence-associated factors, the secretion system is involved in various pathogeneses, including gene or toxic protein transport, adhesion, and environmental stress defense [11, 12]. Despite numerous studies on the pathogenicity and virulence-associated factors of *Campylobacter* species, understanding the virulence traits of *Campylobacter* remains challenging because of their high genetic diversity and frequent intraspecific recombination [13]. High-throughput sequencing-based genomics can help to overcome these limitations by providing detailed information on the structure of genomes and genetic variations of actual bacterial strains, and allowing access to numerous public databases for analysis.

*C. jejuni* has two major types of plasmids: pTet and pVir. The pTet-like plasmid family is the most commonly observed plasmid type in *C. jejuni* and is characterized by the presence of the *tet(O)* gene, which likely contributes to the maintenance of *Campylobacter* plasticity [14]. Second, the pVir-like plasmid, recognized as a virulence plasmid, has been reported to be associated with adhesion processes in the *C. jejuni* 81-176 strain [15]. Additionally, approximately 29% of *C. jejuni* strains isolated from patients with bloody diarrhea were found to carry both pTet and pVir plasmids [16]. Plasmids in *C. jejuni* play an important role in the horizontal spread of antimicrobial resistance (AMR) and pathogenicity, making the understanding of *C. jejuni* plasmids essential for characterizing strain properties.

Poultry and ruminants have been reported to be one of the leading sources of human campylobacteriosis [17, 18]. Food-producing animal farm husbandry is an important reservoir of *C. jejuni* [19]. The possibility of *C. jejuni* transmission from food-animal farms to humans has been proposed through various routes, including the food-chain, direct contact of farm workers with food-animals, and manure excretion from farms into the surrounding environment, such as soils, ponds, and rivers [20]. Previous studies employing methods such as multilocus sequence type (MLST) or pulsed-field gel electrophoresis (PFGE) have faced challenges due to their low discriminatory power, which limits the ability to precisely differentiate and track *C. jejuni* strains [21]. In contrast, studies based on whole-genome sequencing (WGS), with its higher discriminatory resolution, offer a more robust framework for investigating the potential transmission of *C. jejuni* in the food-chain [22]. This approach is even more valuable in modern society, where food distribution systems have become increasingly complex and globalized compared to the past.

In this study, we aimed to elucidate the resistance and virulence mechanisms of *C. jejuni* isolated from chickens and dairy cattle, utilizing WGS-based comparative genomic and phenotypic analyses. To achieve this, we first analyzed AMR and its determinants in *C. jejuni* strains. Second, we investigated virulence factors (VF) and survival-related phenotypes, such as biofilm formation, adhesion ability, and conjugation, and analyzed their associations with genetic traits. Third, we examined the plasmid components of *C. jejuni* strains and assessed their potential for horizontal transfer and chromosomal integration. Finally, we performed WGS-based phylogenetic analysis of *C. jejuni* strains from this study as well as from various sources published in public databases.

## Materials and Methods

### Bacterial Strains

Overall, 94 *C. jejuni* strains isolated from livestock in our previous study were included in the current research [17]. Among the 94 *C. jejuni* isolates, 67 strains were isolated from the manure and farm environments of eight dairy cattle farms, and the remaining 27 strains were isolated from chicken slaughterhouses and retail chicken meat (Table S1). *C. jejuni* strains were isolated following a previously described protocol with some modifications [17]. Briefly, 1 g of each sample was homogenized in 9 ml of Bolton broth containing 5% hemolyzed horse blood and a Bolton broth selective supplement (Oxoid, UK). The samples were microaerobically incubated at 42°C for 48 h. The following day, one loop of broth was streaked onto modified charcoal cefoperazone deoxycholate agar (mCCDA) containing CCDA selective supplement (Oxoid), and the samples were incubated at 42°C for 48 h microaerobically. Next, two to six suspected colonies from the mCCDA plates were picked, subcultured on blood agar, and incubated microaerobically at 42°C for 48 h. To identify *C. jejuni* isolates, multiplex PCR targeting the 16S rRNA gene and *cj0414* and singleplex PCR targeting *hipO* were performed using DNA templates according to methods described previously [17].

### WGS and WGS-Based Comparative Genomic Analysis: Antimicrobial Resistance Determinants and Virulence Factors

All 94 *C. jejuni* strains were used for the WGS-based comparative genomic analysis. Total genomic DNA was extracted using the Nucleospin Microbial DNA Kit (Germany) following the manufacturer’s instructions. Total genomic DNA was sequenced using NextSeq 500 technology (Illumina, USA). The reads were assembled using Unicycler (v0.5.0) and annotated using Prokka (v1.14.5). The WGS data presented in this study have been deposited in the National Center for Biotechnology Information (NCBI) sequence read archive repositories (PRJNA1182330). The assembled contigs were analyzed using bioinformatics tools for the presence of AMR determinants (ResFinder v4.1) [23] and the virulence factor database (VFDB v2019) [24].

### Antimicrobial Susceptibility Test

To test antimicrobial susceptibility, the minimum inhibitory concentrations (MIC) were determined for 88 of the 94 *C. jejuni* strains. Six *C. jejuni* strains were excluded from this analysis because they were identified as viable but nonculturable (VBNC) in the antimicrobial susceptibility test. Susceptibility tests were conducted for seven antimicrobials, including erythromycin, ciprofloxacin, tetracycline, chloramphenicol, gentamicin, azithromycin, and nalidixic acid, using the broth microdilution method with the Sensititre custom plate KRCAMP2 (TREK Diagnostics, USA) and Sensititre CAMPY2 plate (TREK Diagnostic Systems). Resistance to all antimicrobials was determined using an interpretative standard suggested by the National Antimicrobial Resistance Monitoring System (NARMS, https://www.cdc.gov/narms/antibiotics-tested.html). AMR patterns, including multidrug resistance (MDR) patterns, defined as acquired non-susceptibility to at least one agent in three or more antimicrobial categories, were analyzed.

### Biofilm Formation Assay

To test the phenotypic virulence of *C. jejuni* strains isolated from livestock, we tested the biofilm-forming ability of 90 *C. jejuni* strains. Biofilm formation assays were performed according to a previously described protocol, with modifications [25]. Briefly, freshly cultured (42°C, 48 h) *C. jejuni* strains were diluted with fresh MH broth to a McFarland scale of 0.5. Approximately 100 μl of this dilution was added into 96-well microtiter plate (polystyrene) and incubated for 48 h at 42°C in a stationary condition. Each bacterial suspension was inoculated into three wells of a microtiter plate. The growth optical densities (OD) were measured at λ = 595 nm with a SkanIt multiplate reader (Thermo Fisher Scientific, USA). The wells were then washed once with 200 μl of phosphate-buffered saline (PBS), dried for 30 min, and stained with 100 μl of 0.1% crystal violet for 30 min. This was followed by three gentle washes with 200 μl of PBS and air-drying for 1 h. The absorbed dye was solubilized in 100 μl of 30% glacial acetic acid, and the OD were read at 595 nm. The extent of biofilm formation was calculated using the following formula: SBF = $\frac{\left(AB-CW\right)}{G}$, where SBF is the specific biofilm formation index, AB is the OD595 of the stained bacteria, CW is the OD595 of the stained control wells containing absolute media without bacteria, and G is the OD595 corresponding to cell growth in the media. An SBF value > 0.5 was suggested as positive biofilm formation. *E. coli* ATCC 25922 was used as the positive control, and the culture medium was used as the negative control.

### Cell Adhesion Assay

The HT-29 human intestinal cell line, obtained from the Korean Cell Line Bank, was used to test the cell adhesion ability of *C. jejuni* strains isolated from livestock. The adhesion assay and cell maintenance were performed as described by Haddad *et al*. (2010) with slight modifications [26]. Briefly, cultured cells were dissociated from flasks using 0.25% Trypsin-EDTA solution (Gibco) and approximately 4 × 10^4^ eukaryotic cells were seeded into each well of a 96-well cell culture plate and incubated for 3 days at 37°C in humidified atmosphere at 5% of CO_2_. The cells were washed twice with 1x PBS and each well was inoculated with a suspension of approximately 1.5 × 10^7^ CFU of the *C. jejuni* strain. To evaluate the number of adhered *C. jejuni*, the infected monolayers were incubated for 2 h at 37°C in a humidified 5% CO_2_ incubator and rinsed twice with 1x PBS. The cell monolayer was dissociated using 0.25% trypsin-EDTA solution for 10 min. Ten-fold serial dilutions of the lysates were plated on mCCDA plates and incubated at 42°C for 48 h under a microaerobic condition, and colonies were enumerated to determine the number of cell-associated bacteria. The experiments were performed using three biological replicates.

### Comparative Genomic Analysis of the Mobile Genetic Element (MGE) of *C. jejuni* Strains

To identify the MGE of *C. jejuni* strains, the Graphical Fragment Assembly (GFA) obtained through Unicycler was visualized using the Bandage program and then analyzed [27]. Unicycler uses SPAdes to assemble the Illumina reads into an assembly graph and attempts assemblies at a wide range of *k-mer* sizes, evaluating the best graph for each [27, 28]. Unicycler builds bridges between single-copy contigs using path information in the SPAdes assembly. Considering that the chromosome size of *C. jejuni* is 1.7 Mbp (1.567 Mbp to 2.127 Mbp in NCBI genome RefSeq database) on average [29], if the summation of bridged-contigs and scaffolds exceeds 1.0 Mbp, the corresponding contig was assumed to a chromosome. Additionally, small genetic compartments with circular shapes without dead ends were considered as plasmids (Fig. S1). The presence of AMR determinants and virulence genes was analyzed using ResFinder (v4.1) [23] and VirulenceFinder (v2.1) [30], respectively. Comparative genome visualization was performed using easyfig (v2.2.3) [31] and the GView server [32].

### Conjugation Assay

To assess the horizontal transferability of MGE in *C. jejuni* strains, we performed a conjugation assay based on a previously described protocol with slight modifications [33]. This assay used streptomycin-resistant wild-type *C. jejuni* CJ518 as the recipient strain. Of the 18 plasmid types, 16 strains representing 15 plasmid types were selected as donors, excluding pTet_SNU14 and pTet_SNU17 because of their streptomycin resistance, and pVir_SNU01 because of the absence of resistance markers. Transconjugants were selected on Mueller-Hinton agar plates (Sigma-Aldrich) containing 16 mg/l streptomycin and 8 mg/l tetracycline. The presence of the *pgm* gene with an allele number of 140, part of the MLST scheme, in the transconjugants was confirmed via Sanger sequencing (pgm-S3:5'-GCT TAT AAG GTA GCA CCT ACT G-3', pgm-S2: 5'-TCC AGA ATA GCG AAA TAA GG-3'). In addition, *tet(O)* (F: 5'-TGG AGC GTC AAA GGG GAA TC-3', R: 5'-TTA CCG CAT CCC ACT GTT CC-3') and *virB4* (F: 5'-AAG GCA AAC AAT TTG GCT GG-3', R: 5'-AGC CAC TTC CAA GCT TCA TCA-3') were identified by PCR to confirm that the plasmid and T4SS gene cassette were transferred. The primer sets used in this study were designed using the NCBI Primer-BLAST program [34].

### MLST and Core-Genome Multi-Locus Sequence Types (cgMLST)-Based Genetic Relatedness Analysis

To examine the genetic relatedness between *C. jejuni* strains from various sources, we conducted in-silico MLST [35] and cgMLST [36] First, we conducted in-silico MLST for 94 *C. jejuni* strains isolated from this study, and the minimum spanning tree (MST) was constructed based on the allelic profiles of seven MLST housekeeping genes utilizing BioNumerics software (v6.6, Applied Maths N.V., Belgium). Next, for the two most prevalent ST-21 and ST-42 strains, we performed cgMLST on *C. jejuni* strains isolated in this study and on *C. jejuni* strains whose WGS data and metadata (host, country, and year of isolation) were uploaded to the NCBI Pathogen Isolate Browser. For visualization of cgMLST results, an MST and unweighted pair group method with arithmetic mean (UPGMA) dendrogram was constructed based on allelic profiles of 1,343 cgMLST loci utilizing BioNumerics software (v6.6). Isolates showing ≥ 95% similarity in the UPGMA dendrogram were assigned to phylogenetic clades, and three clades (I,II, and III) were identified for each of the ST-21 (Fig. S2), ST-42 strains (Fig. S3), and ST-48 strains (Fig. S4).

### Statistical Analysis

All statistical analyses were conducted using a simple random sampling procedure with the Statistical Package for the Social Sciences (SPSS) program (v27.0, IBM SPSS Statistics for Windows, USA). For comparative analysis of AMR, biofilm formation, cell adhesion rate, and presence of AMR and VF, Fisher’s exact test was used to calculate the odds ratios (OR) and 95% confidence intervals setting cattle-derived strains as a reference. Where zeros caused problems in calculating the OR in the GEE analysis, Fisher’s exact test was conducted by adding 0.5 to each cell [37]. Spearman’s correlation test was performed to evaluate the correlation between the presence of AMR determinants and phenotypic AMR, and between the presence of VF and biofilm formation/cell adherence ability.

## Results

### Phenotypic and Genotypic Antimicrobial Resistance of *C. jejuni* Strains from Livestock

**Fig. 1:**
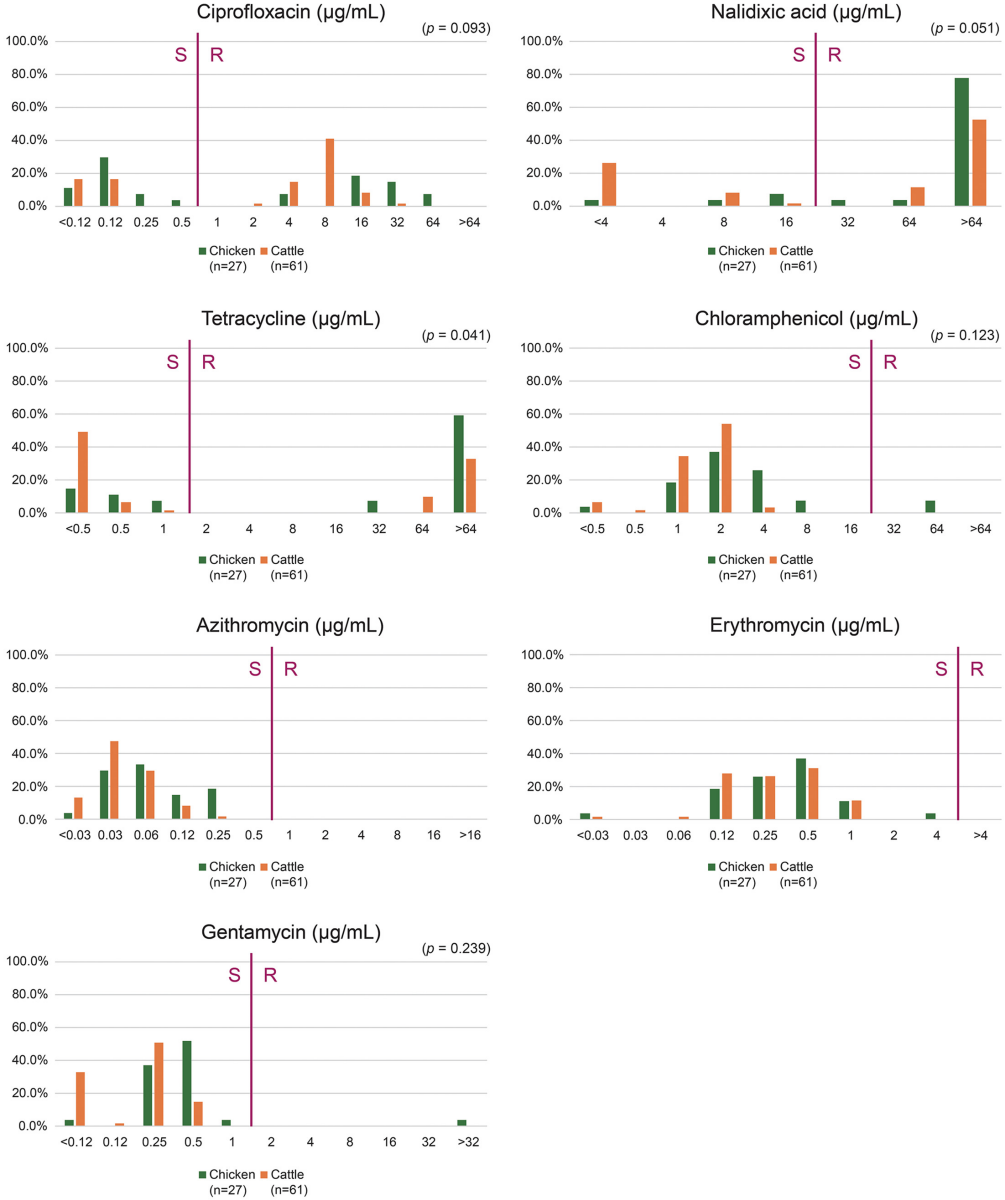
Antimicrobial resistance phenotypes of *C. jejuni*. Minimum inhibitory concentration assay results for the antimicrobial resistance phenotypes of *C. jejuni* against seven antimicrobial agents. The statistical significance (*p*-value) for each antibiotic was calculated using a 2 × 2 chi-square test for cattle/chicken and susceptible/resistant (S/R) categories. Abbreviation. S: susceptible, R: resistance

Among the 88 *C. jejuni* strains, the resistance rate to nalidixic acid was 70.5% (62/88), that to ciprofloxacin was 61.4% (54/88), that to tetracycline was 50.0% (44/88), that to chloramphenicol was 2.3% (2/88), and that to gentamycin was 1.1% (1/88) (Fig. 1). Resistance to macrolides, including erythromycin and azithromycin, is yet to be identified. Compared to cattle-derived strains, chicken-derived strains showed significantly higher resistance rate to tetracycline (OR: 2.7, 95% CI: 0.99–10.59, *p* < 0.05, Table 1), marginally higher resistance rate to nalidixic acid (OR: 3.2, 95% CI: 0.18–1.14, *p* = 0.051), and marginally lower resistance rate to ciprofloxacin (OR: 0.5, 95%CI: 0.18–1.14, *p* = 0.093). The three chicken-derived MDR strains were resistant to chloramphenicol-tetracycline-quinolones (CJ502 and CJ505) and tetracycline-gentamycin-quinolones (CJ509).

In the WGS-based analysis of AMR determinants, the most prevalent AMR gene was *bla*OXA-61 (67.0%, 63/94), followed by *gyrA*(T86I) (61.7%, 58/94) and *tet(O)* (29.8%, 28/94) (Table 2). In the correlation analysis, significant positive correlations were identified between the AMR determinants and phenotypic resistance to aminoglycosides (*ant(6)-Ia*, *aph(2”)-Ih*, and *aph(3’)-III*), tetracyclines (*tetO*), quinolones (*gyrA*(*T86I*)), and phenicols (*cat*, Spearman’s correlation, *p* < 0.05, Table S2).

### Virulence Factors of *C. jejuni* Strains from Livestock

In the WGS-based virulence factor analysis, 119 virulence factors and 11 distinct VF families were identified in 94 *C. jejuni* strains. More than 90% prevalence was identified for 10 VF families, including CadF (100.0%, 94/94), Capsule (100.0%, 94/94), Cia (100.0%, 94/94), Flagella (100.0%, 94/94), Pse (100.0%, 94/94), CDT (97.9%, 92/94), PEB1 (97.9%, 92/94), LOS (95.7%, 90/94), Capsule biosynthesis and transport (94.7%, 89/94), and JlpA (94.7%, 89/94) (Table S3). The lowest prevalence was identified for major outer membrane protein (MOMP, 10.6%, 10/94). In the comparative analysis between sources, no significant differences were identified between cattle- and chicken-derived strains at the VF family level.

Out of 119 VF, a 100% prevalence was observed for 18 VF, including Campylobacter adhesion to fibronectin F (*cadF*), Campylobacter invasion antigen B (*ciaB*), 10 Flagella-associated VFs, and 6 Pse-associated VFs (Table S3). In contrast, *flab* of the Flagella family, *pseI* of the Pse family, and seven T4SS VF were detected in only one strain. Compared to cattle-derived strains, chicken-derived strains showed a significantly higher prevalence of capsule-associated *kpsC*, capsule biosynthesis, transport-associated *Cj1419c* and *Cj1420c*, *ciaC*, and flagella-associated *fliI*, *motA*, and *motB* (Fisher’s exact test, *p* < 0.05, Table 3). In contrast, the prevalence of two flagella-associated VF, *pseB* and *pseC*, was significantly higher in the cattle-derived strains than in the chicken-derived strains (Fisher’s exact test, *p* < 0.05).

### Biofilm Formation and Cell Adherence Ability of *C. jejuni* Strains from Livestock

Among 91 *C. jejuni* strains, 31.9% (29/91) of *C. jejuni* strains exhibited biofilm formation and 7.7% (7/91) of *C. jejuni* strains showed strong biofilm formation. The chicken-derived strains (30.8%, 8/26) and cattle-derived strains (32.3%, 21/65) exhibited similar biofilm formation abilities (Fisher’s exact test, *p* > 0.05). Among the 119 VFs identified from *C. jejuni* strains in this study, the prevalence of 3 VF, specifically *pseH* (coef: 0.369, *p* < 0.001, Spearman’s correlation test), *flgJ* (coef: 0.247, *p* < 0.05), *pseB* (coef: 0.218, *p* < 0.05), was positively correlated with biofilm formation.

In the cell adhesion assay, the average adhesion rate was 52.4% to the HT29 cell line. Chicken-derived strains (59.3%) exhibited significantly higher adhesion ability to HT29 cells compared with cattle-derived strains (50.2%, *t*-test, *p* < 0.05). Among 119 VF, the prevalence of 5 VF, *Cj1417c* (coef: 0.265, *p* < 0.05, Spearman’s correlation test), *Cj1420c* (coef: 0.296, *p* < 0.05), *flaD* (coef: 0.266, *p* < 0.05), *flgI* (coef: 0.258, *p* < 0.05), and *flgJ* (coef: 0.240, *p* < 0.05), were positively correlated with the adherence rate to HT-29 cells.

### Genetic Characterization of the MGE of *C. jejuni* Strains

**Fig. 2:**
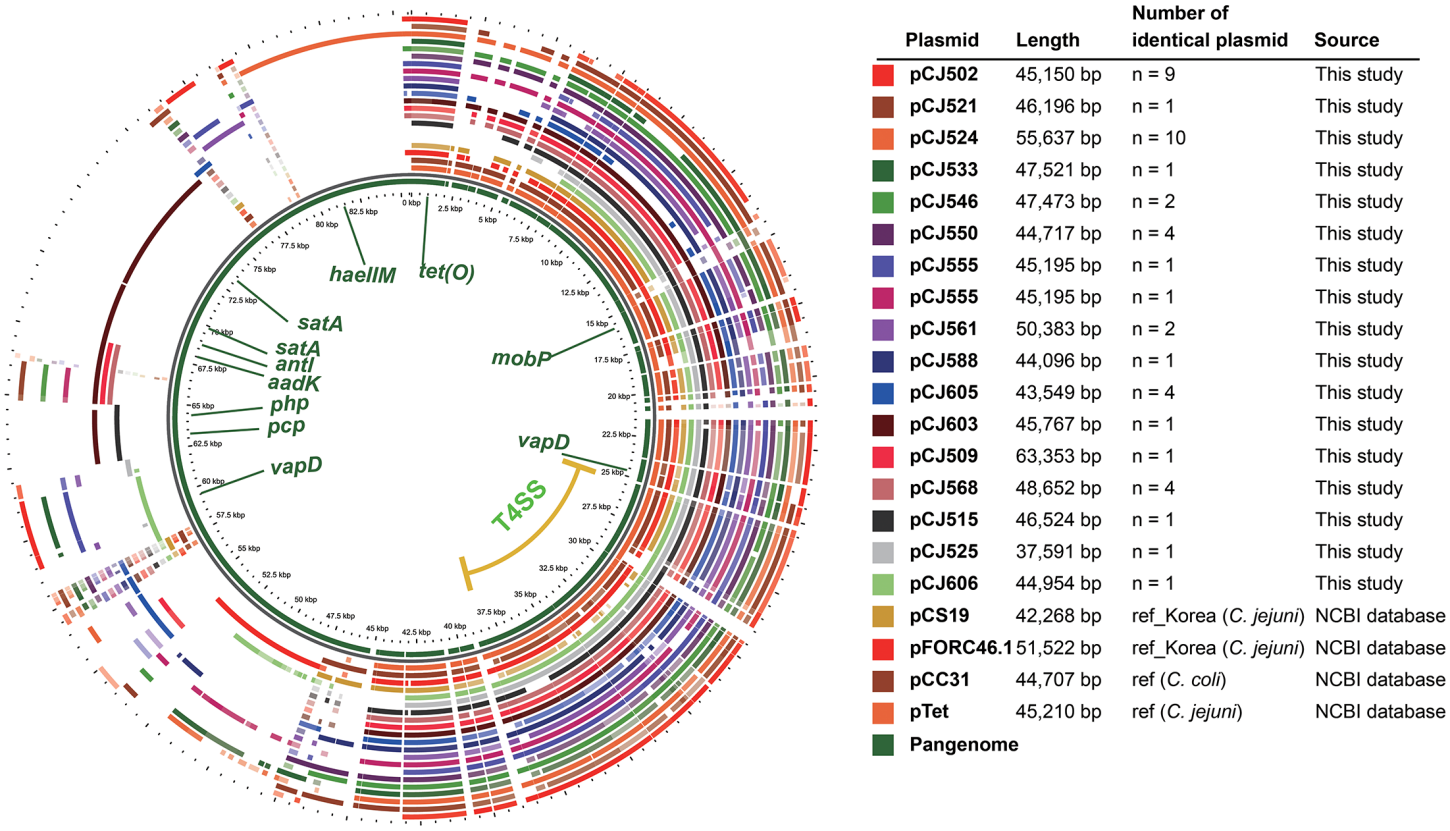
Comparative genomic analysis of the pTet family plasmid in this study and NCBI databases. Each circle represents the gene in each plasmid, with the innermost circle indicating the pan-genome. The plasmids pCS19, pFORC46.1, pCC31, and pTet were used as references.

**Fig. 3:**
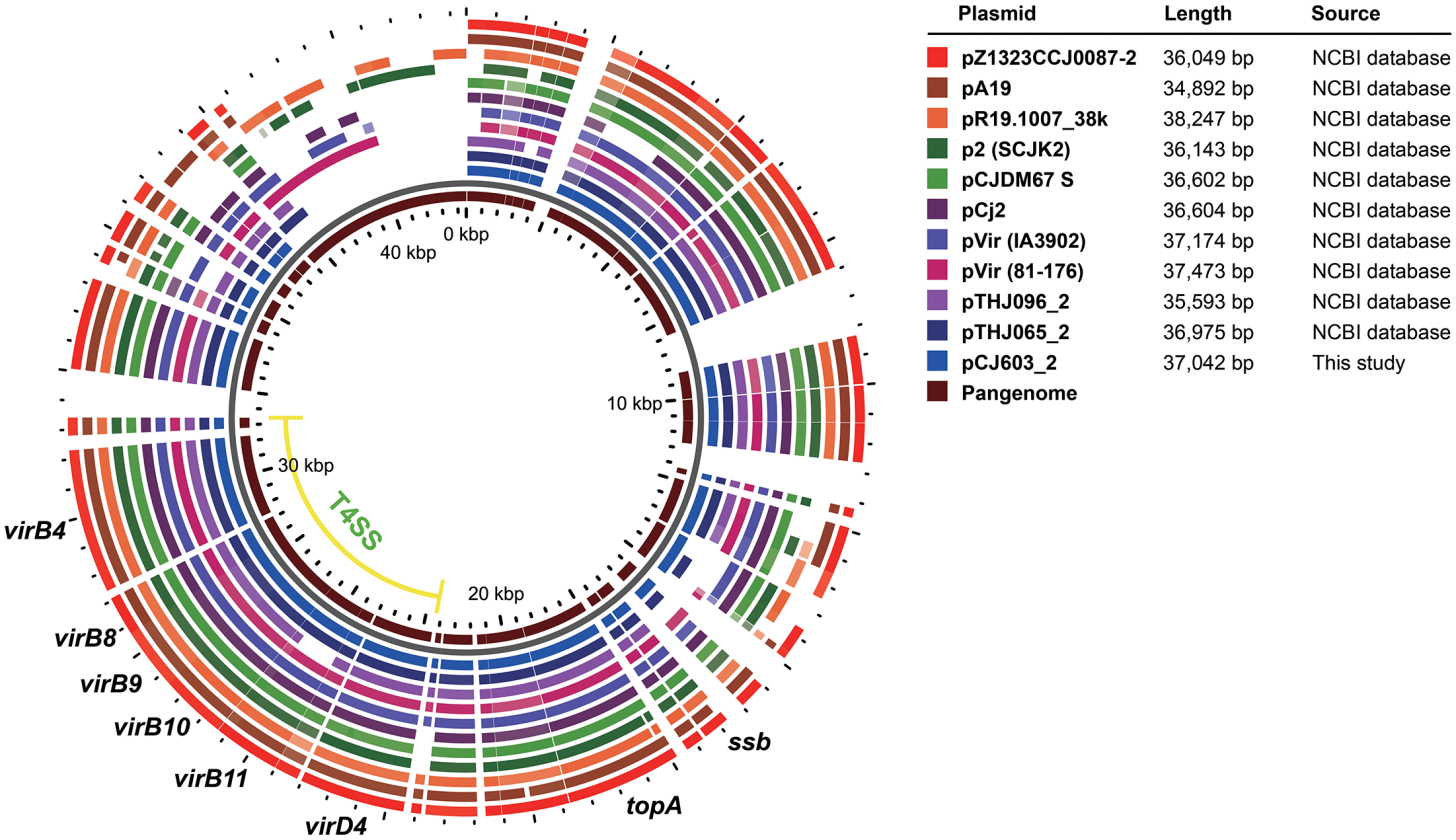
Comparative genomic analysis of the pVir family plasmid in this study and NCBI databases. Each circle represents the gene in each plasmid, with the innermost circle indicating the pan-genome. The pCJ603_2 plasmid was identified in this study, whereas the remaining ten plasmids are pVir family plasmids deposited in the NCBI nucleotide database.

Given the established role of plasmids in AMR and virulence in *Campylobacter* [38], we conducted a plasmid analysis of *C. jejuni* strains in this study. Of the 94 *C. jejuni* strains, 45 strains (47.9%) carried the pTet-family, of which one strain, CJ603, carried an additional pVir-family plasmid. In total, 17 types of the pTet family (length: 39,591–63,353 bp) were identified (Fig. 2 and Table S4). The most dominant type was pTet_SNU01 type (20.0%, 10/50), followed by pTet_SNU02 (18.0%, 9/50) and pTet_SNU12 (10.0%, 5/50). Eleven plasmid types were identified in single isolates. Four types, including pTet_SNU01, pTet_SNU02, pTet_SNU12, and pTet_SNU13, were identified in both cattle- and chicken-derived *C. jejuni* strains. In the comparative genomic analysis with the NCBI reference genome, the pCJ550 (pTet_SNU04 type) plasmid showed high genetic similarity (99.9%, ClustalW) to the plasmid pCC31 of the reference *Campylobacter coli* strain. All pTet family plasmids carried the tetracycline resistance gene *tet(O)*, except for pTet_SNU15 and pTet_SNU16 (Table S4). The aminoglycoside resistance genes, *ant(6)-Ia*, *aph(3')-III*, *aph(2'')-Ih*, and *ant(6)-Ia*, were identified from pTet_SNU14. All pTet family plasmids carried the T4SS (VirB2-VirB4-VirB5-VirB6-VirB7-VirB8-VirB9-VirB10-VirB11-VirD4; Fig. S5) and the virulence-associated protein D coding gene, *vapD*. For the conjugation assay, pTet_SNU03 and pTet_SNU12 were horizontally transferred to the donor strain. The pVir_SNU01 (37,042 bp) carried T4SS (VirB4-VirB7-VirB8-VirB9-VirB10-VirB11-VirD4), with no AMR determinants or VF identified on this plasmid (Fig. 3). No genetic similarity was identified between the nucleotide sequences of the T4SS in the pTet-family and pVir-family plasmids. Comparative genomic analysis of the pVir_SNU01 type plasmid pCJ603_2 and ten other pVir plasmids revealed a high degree of homology among the plasmids, with very few exceptions. Notably, deletion of *virB11* was observed in the pTH096_2 plasmid.

In four *C. jejuni* strains, CJ505, CJ528, CJ542, and CJ604, genetic regions highly homologous with pTet-family plasmids were identified on their bacterial chromosomes. Comparative genomic analysis of the CJ542 strain showed that a plasmid fragment (44,790 bp) was integrated between *fokIM* and a hypothetical protein on the CJ542 chromosome (Fig. S6). In the comparative genomic analysis the *C. jejuni* D4860 pD4860-1 plasmid, the pTet plasmid fragment of CJ542 showed high sequence homology of 99.9% similarity in amino acids. The cleavage site of plasmid fragment of CJ542 was between *virB2* and *cpp29*, which are known T4SS components.

### MLST and cgMLST-Based Phylogenetic Analysis of *C. jejuni* Strains

**Fig. 4:**
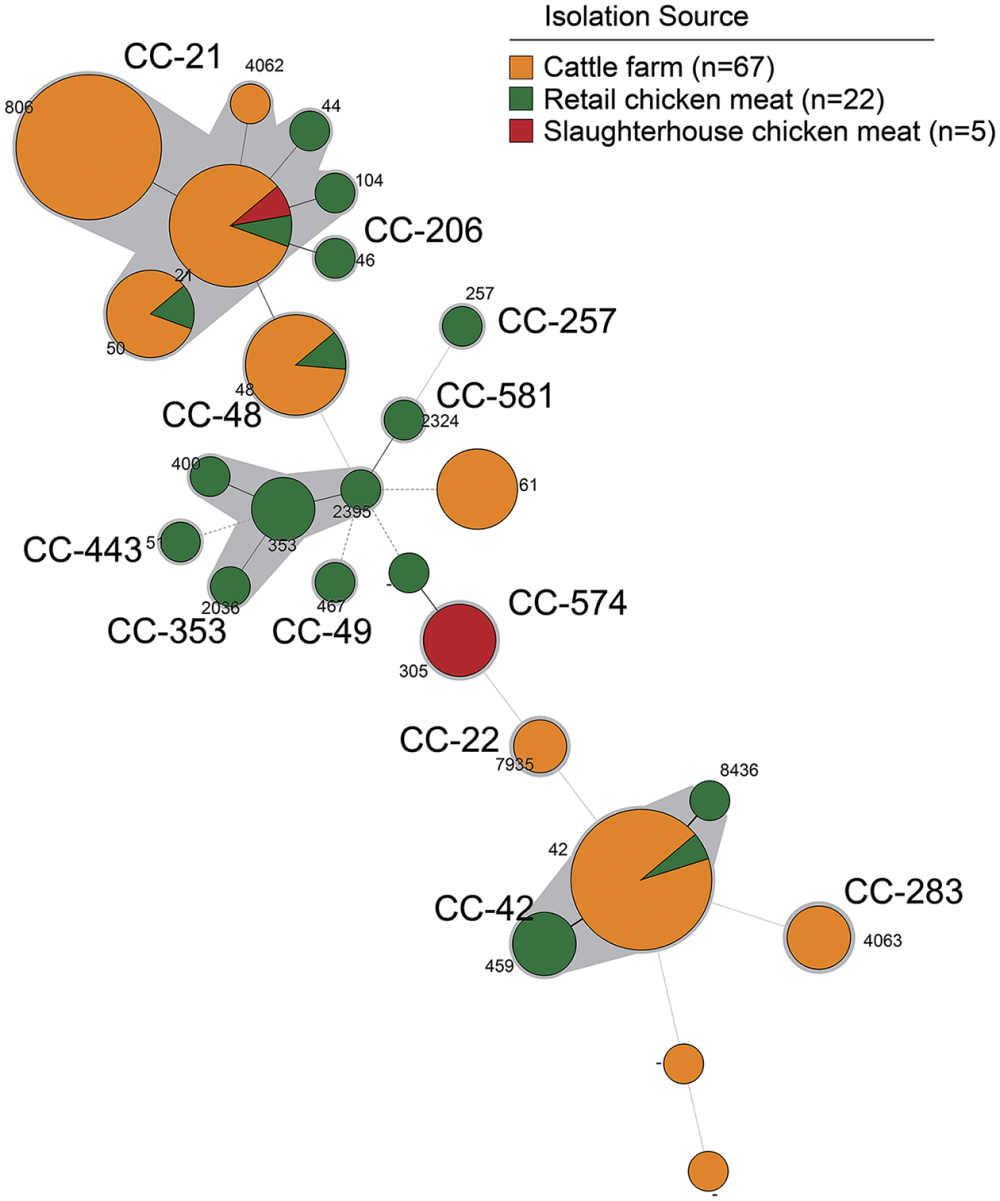
Minimum spanning trees based on the allelic profiles of 26 multi-locus sequence types. Each node within the tree represents a single ST. The length of branches between each node represents the number of different alleles (out of seven MLST genes) that differ between the two linked nodes/Sequence type (ST). The node size is proportional to the frequency of the sequence type occurrence, and the colors indicate isolation sources.

In the MLST phylogenetic analysis, 94 *C. jejuni* strains matched 23 STs from the reference PubMLST database, and three isolates did not match the database because of an untypeable locus combination or alleles that represented novel STs (Table 4 and Fig. 4). The 23 ST were linked to 12 CC. The isolates were distributed widely across different CC, with the majority belonging to CC21 (39.4%, 37/94), CC42 (21.3%, 20/94), and CC48 (8.5%, 8/94). The most prevalent ST types were ST-806 (17.0%, 16/94) and ST-42 (17.0%, 16/94), followed by ST-21 (12.7%, 12/94), ST-48 (8.5%, 8/94), and ST-50 (6.3%, 6/94). Four ST types, ST-42, ST-21, ST-48, and ST-50, were identified in both the cattle- and chicken-derived *C. jejuni* strains. For cattle-derived strains, the most prevalent clone type was ST-806 (CC21, 16/67, 23.9%), followed by ST-42 (CC42, 15/67, 22.4%) and ST-21 (CC21, 10/67, 14.9%). For chicken-derived strains, the most prevalent clone type was ST-305 (CC574; 4/27, 14.8%), followed by ST-459 (CC42; 3/27, 11.1%), ST353 (CC353; 3/27, 11.1%), and ST-21 (CC21; 2/27, 7.4%).

**Fig. 5:**
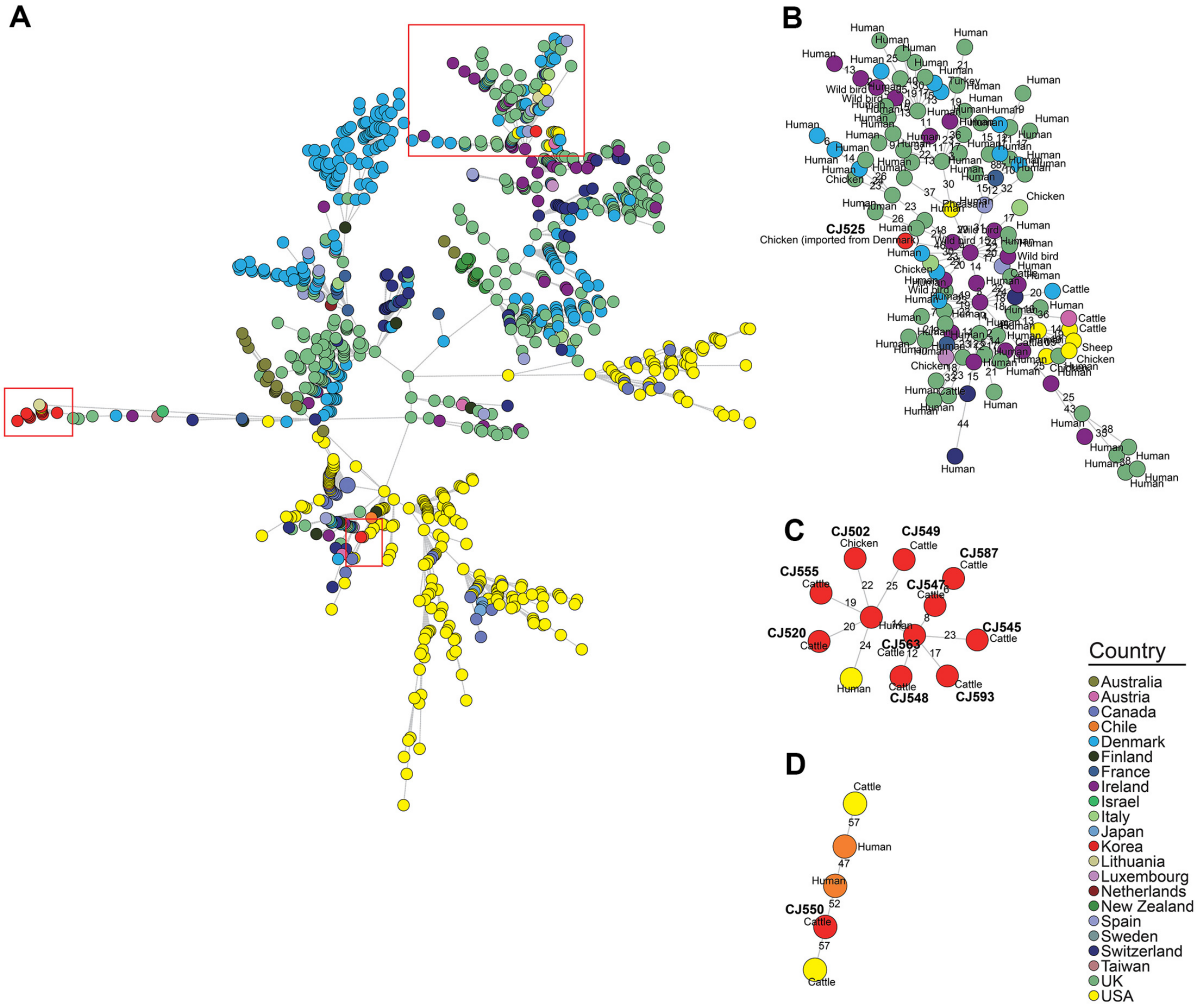
Minimum spanning tree based on the cgMLST allelic profiles of the MLST ST-21 *C. jejuni* strains. Isolates from this study and *C. jejuni* strains belonging to MLST ST-21 from the NCBI isolate browser were used for analysis. Each node represents a different cg-MLST. The length of the branches between each node indicates the number of alleles (out of 1,343 cgMLST genes) that differ between the two linked nodes. The color of each node represents the country from which the strain was isolated. (**A**) The minimum spanning tree (MST) for all strains belonging to ST-21. (**B**), (**C**), and (**D**) Separate MST for phylogenetic clades I, II, and III, which were defined based on 95% similarity criteria in the UPGMA dendrogram.

**Fig. 6:**
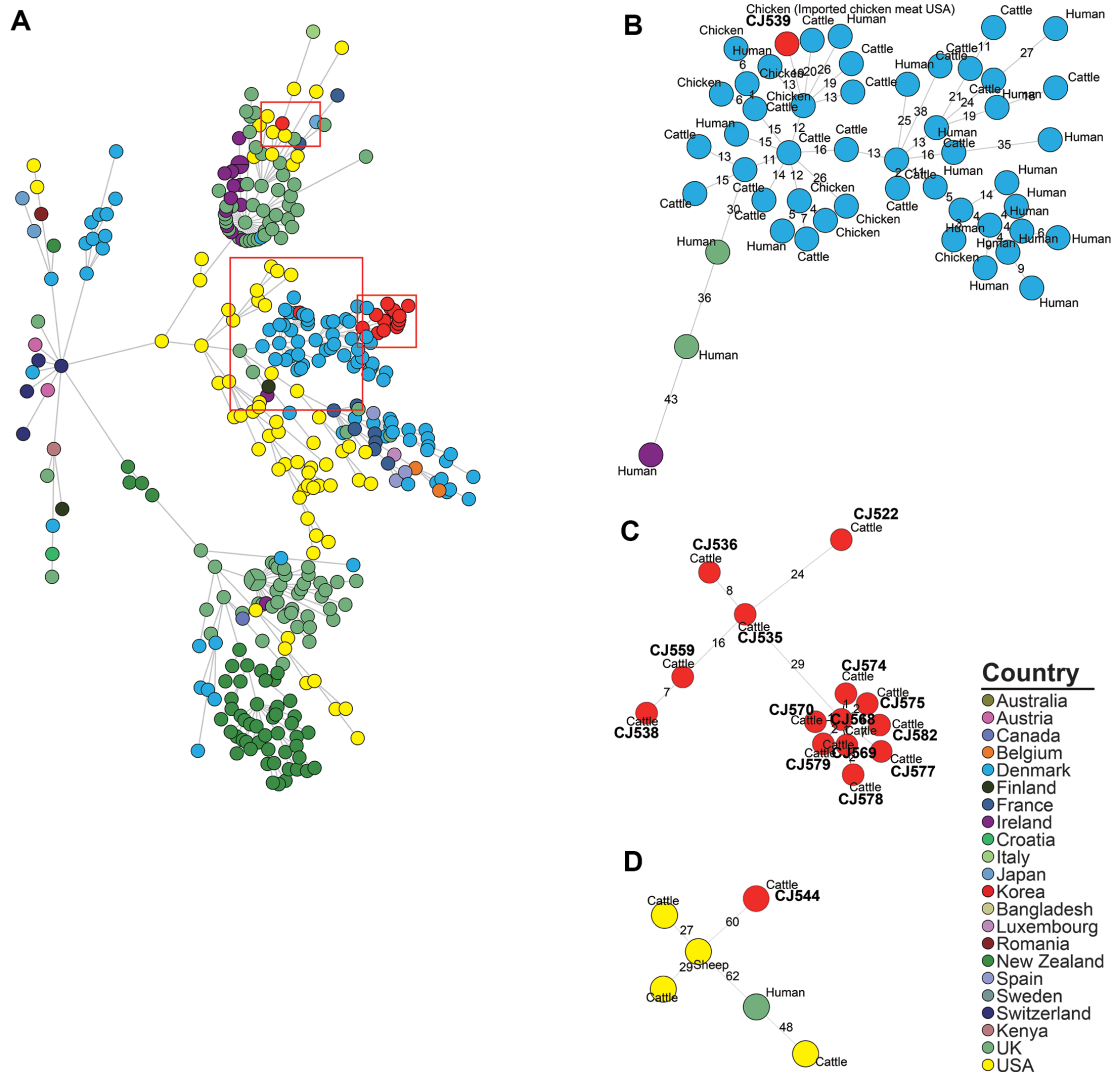
Minimum spanning tree based on the cgMLST allelic profiles of the MLST ST-42 *C. jejuni* strains. Isolates from this study and *C. jejuni* strains classified as MLST ST-42 in the NCBI isolate browser were included in the analysis. Each node represents a distinct cg-MLST type. The branch lengths connecting each node reflect the number of different alleles (out of 1,343 cgMLST genes) that vary between the two connected nodes. The color of each node indicates the country of origin of the isolated strain. (**A**) The minimum spanning tree (MST) for all strains categorized under ST-42. Meanwhile, (**B**), (**C**), and (**D**) depict separate MST for the phylogenetic clades I, II, and III, defined based on a 95% similarity threshold in the UPGMA dendrogram derived from the cgMLST allele profiles.

**Fig. 7:**
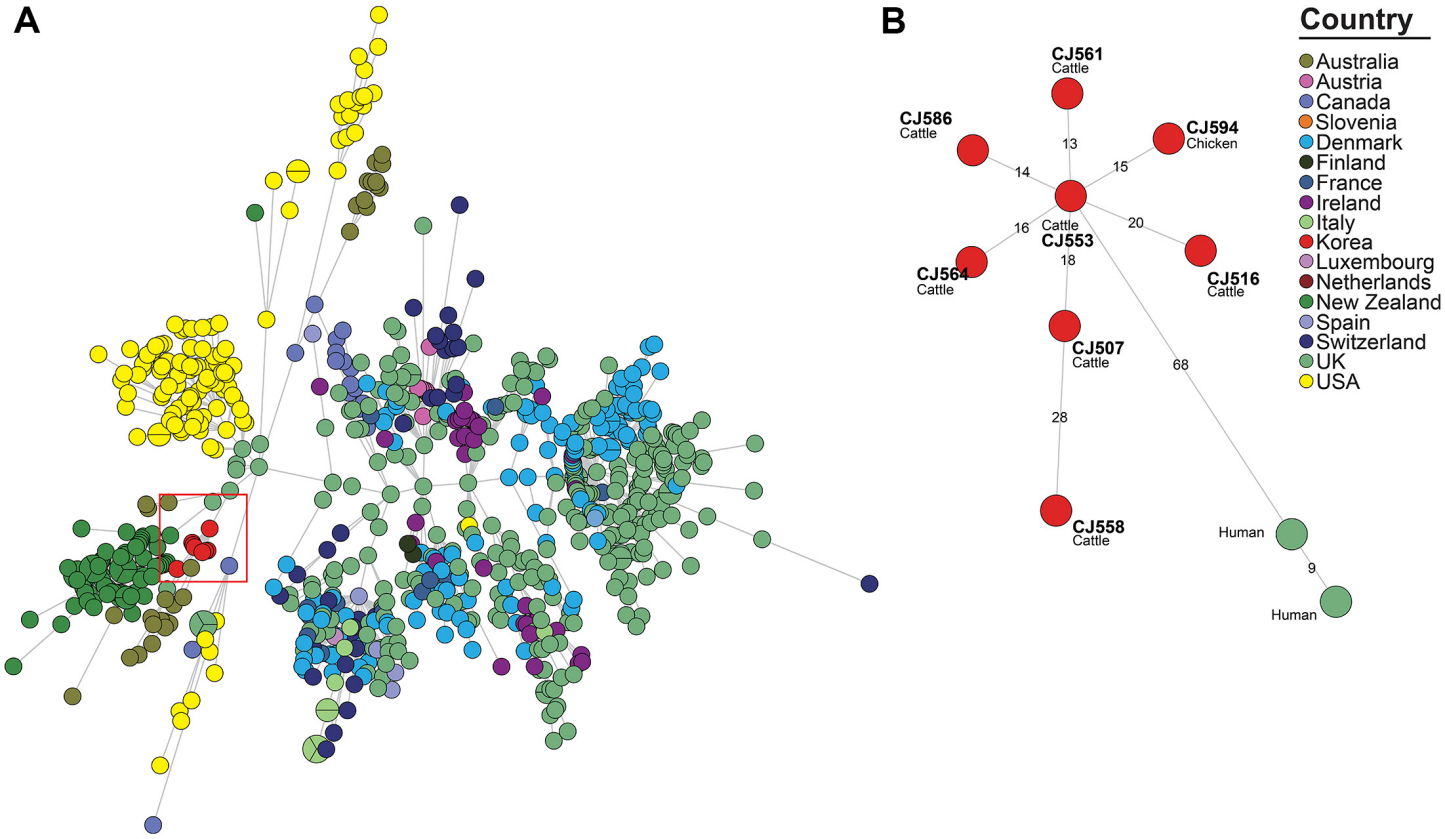
Minimum spanning tree based on the cgMLST allelic profiles of the MLST ST-48 *C. jejuni* strains. Isolates from this study and *C. jejuni* strains classified as MLST ST-48 in the NCBI isolate browser were included in the analysis. Each node represents a distinct cg-MLST type. The branch lengths connecting each node reflect the number of different alleles (of 1,343 cgMLST genes) that vary between the two connected nodes. The color of each node indicates the country of origin of the isolated strain. (**A**) The minimum spanning tree (MST) for all strains categorized as ST-48. Meanwhile, (**B**) depicts separate MST for the ST-48 phylogenetic clade I, defined based on a 95% similarity threshold in the UPGMA dendrogram derived from the cgMLST allele profiles.

To examine the genetic relatedness between the cattle- and chicken-derived strains isolated in this study and the *C. jejuni* strains isolated from various sources, we conducted cgMLST-based phylogenetic analysis. In the cgMLST-based MST (Figs. 5, 6 and 7), 12 ST-21 *C. jejuni* strains isolated in this study assigned to three phylogenetic clades (I, II, and III) based on a similarity threshold of over 95%. (Fig. S2). Ten *C. jejuni* strains (one chicken- and nine cattle-derived strains) were assigned to phylogenetic clade I, consisting of GCA_032735145.1 (Human, Korea, 2012) and GCA_004845505.1 (Human, USA, 2014). CJ525 (imported chicken meat from Denmark, 2009) was assigned to phylogenetic clade II, consisting of GCA_001229025.1 (Human, UK, 2011), GCA_033218075.1 (Human, Denmark, 2019), and GCA_033224905.1 (Turkey, Denmark, 2016). CJ550 (cattle, 2012) was assigned to phylogenetic clade III, consisting of GCA_033041535.1 (Human, Chile, 2020) and GCA_022398905.1 (Cattle, USA, 2017). Sixteen ST-42 *C. jejuni* strains isolated in this study were assigned to three phylogenetic clades (I, II, and III; (Fig. S3). Fourteen cattle-derived ST-42 *C. jejuni* strains were assigned to phylogenetic clade I, which was constructed without other source-derived strains. CJ539 (Imported chicken meat from USA, 2006) was assigned to phylogenetic clade II, consisting of GCA_033315885.1 (Cattle, Denmark, 2016), GCA_033308595.1 (Chicken, Denmark, 2016), and GCA_033307355.1 (Human, Denmark, 2015). One cattle-derived strain CJ544 (2012) was assigned into phylogenetic clade III, consisting of GCA_012455925.1 (Cattle, USA, 2020), GCA_032195985.1 (Sheep, USA, 2007), and GCA_012446015.1 (Cattle, USA, 2019). Seven cattle-derived and one chicken-derived ST-48 strains from this study were grouped into ST-48 phylogenetic clade I, along with two human isolates collected from the United Kingdom in 2018 (GCA_007936905.1, GCA_007942325.1) (Fig. S4).

## Discussion

*C. jejuni*, a leading cause of bacterial foodborne diarrheal diseases, has been reported to be commonly transmitted through improperly handled chicken meat and unpasteurized milk. Our previous research reported that chickens and cattle are major reservoirs of *Campylobacter*, with certain clonal types, such as ST-21 and ST-42, frequently detected in these animals, as well as in humans. In addition, *C. jejuni* isolates from chicken and cattle exhibit a high prevalence of virulence factors critical to the pathogenicity of *Campylobacter*, including T4SS and T6SS, which play crucial roles in both virulence and AMR. In this study, we aimed to elucidate the resistance and virulence mechanisms of *C. jejuni* in livestock using comparative genomic and phenotypic analyses.

The prevalence of antimicrobial-resistant *Campylobacter* spp. has increased, representing a growing public health concern worldwide. In particular, fluoroquinolone-resistant *Campylobacter* is listed as one of the 12 critically important antimicrobial resistant priority pathogens by the WHO [39]. The indiscriminate use of antibiotics in the livestock industry is an important risk factor for the emergence of resistant bacteria in food animals [40]. According to the annual report of the Korean Ministry of Food and Drug Safety, 25,000 kg of fluoroquinolones are used annually in the poultry industry, 9,000 kg in the swine industry, and 2,000 kg in the cattle industry [41]. In this study, 70.5% and 61.4% of *C. jejuni* strains exhibited resistance to nalidixic acid and ciprofloxacin, respectively. Interestingly, the resistance rate to nalidixic acid was significantly higher in the chicken-derived strains. In contrast, although not statistically significant, a higher ciprofloxacin resistance rate was identified in the cattle-derived strains. Fluoroquinolones target DNA gyrase and topoisomerase IV, disrupting DNA replication [42, 43]. In *Campylobacter* spp., resistance primarily arises from point mutations in the quinolone resistance-determining region of the GyrA subunit of DNA gyrase [44, 45]. In WGS-based comparative genomic analysis, *C. jejuni* strains had a higher prevalence of AMR determinants, including *gyrA* (T86I) point mutations. *C. jejuni* with a *gyrA* mutation exhibits significantly high resistance to quinolone-class antimicrobials [33]. Consequently, this mutation often leads to treatment failure with fluoroquinolones, antimicrobial agents commonly used to manage human infections [46]. As a result, prolonged treatment with higher doses of alternative antimicrobials becomes necessary, which may increase the risk of infection spread in both healthcare settings and the broader community. Nalidixic acid, a first-generation quinolone, often confers resistance through single mutations in the *gyrA* gene, often Thr-86-Ile [47]. Meanwhile, Ciprofloxacin, a second-generation fluoroquinolone, was less susceptible to single point mutations that led to high levels of resistance against first-generation quinolones and required additional mutations to achieve similar resistance levels, which can lead to slower development of high-level resistance compared to nalidixic acid [48]. Broilers are marketed at 30–35 days of age, whereas dairy cattle are typically raised for 4–6 years [49]. This suggests that dairy cattle may be exposed to fluoroquinolone antibiotics for longer periods, which could explain the higher ciprofloxacin resistance rate of cattle-derived strains.

In WGS-based comparative genomic analysis, *C. jejuni* strains had a higher prevalence of AMR determinants, including *blaOXA-61*, *tet(O)*, and *rpsL(K43R)* mutations. OXA-61 is prevalent among *Campylobacter* spp. of veterinary origin and is associated with increased resistance to ampicillin, co-amoxiclav (amoxicillin and clavulanic acid), and penicillins [50]. In Campylobacter, various tet genes, including *tetA*, *tetB*, *tetC*, *tetL*, and *tet(O)*, confer tetracycline resistance, with *tet(O)* being the most prominent type [51]. The *tet(O)* gene in *Campylobacter* encodes a ribosomal protective protein that disrupts tetracycline binding by attaching to the ribosome and causing structural changes [52]. The *rpsL*(K43R) mutation in *Campylobacter* causes resistance to streptomycin by altering the structure of the 30S ribosomal subunit and the streptomycin-binding site on the ribosome [53]. These antimicrobial agents have been used extensively for disease management and treatment in livestock, particularly cattle, which are the major natural hosts of *C. jejuni* [41]. This continuous exposure to antibiotics involves the selection of bacteria and is enhanced by their ability to acquire different resistance factors from surrounding bacteria, leading to the emergence of modified strains with diverse resistance traits [54]. The high prevalence of various AMR determinants in *C. jejuni* strains in livestock poses a significant threat by potentially undermining future antibiotic treatment recommendations and protocols, highlighting the need for increased vigilance and careful monitoring.

Biofilms provide a protective environment against pathogens such as *C. jejuni* [55]. As a microaerophilic bacterium, *C. jejuni* can minimize its exposure to aerobic conditions by forming biofilms, allowing it to survive longer and spread more effectively throughout the environment. Adhesion is a key step in bacterial colonization, and a higher rate of adhesion to eukaryotic cells is associated with an increased potential for colonization and invasion, which in turn may enhance pathogenicity [56]. In this study, 31.9% of *C. jejuni* strains showed biofilm formation ability, with 7.7% of *C. jejuni* strains showing strong biofilm formation ability. Biofilm formation positively correlated with the presence of *pseH*, *pseB*, and *flgJ*. Pseudaminic acid (Pse) contributes to biofilm formation by enhancing the adhesion properties of bacterial surfaces, thereby strengthening the biofilm matrix [57]. The adhesion assay revealed that *C. jejuni* strains exhibited a robust adherence to human colon cell-lines HT29, with an adherence rate of 53.6%. Cell attachment positively correlated with the presence of *Cj1417c*, *Cj1420c*, *flaD*, *flgI*, and *flgJ*. *Cj1417c* and *Cj1420c* are involved in capsule biosynthesis, which is crucial for the ability of the bacterium to adhere to surfaces and is a key factor in host colonization. The *pseB* and *pseC* genes, identified at significantly higher levels in cattle-derived *C. jejuni*, are associated with flagellar assembly [58]. The thick intestinal mucus layer in cattle exerts selective pressure for robust flagellar systems, which are critical for bacterial motility and adhesion [59]. Consequently, this dense and active mucus environment in cattle likely drives the prevalence of virulence factors such as *pseB* and *pseC*. This suggests that the unique environmental conditions in cattle gastrointestinal tracts may shape the genetic landscape of *C. jejuni*, emphasizing the role of host-specific adaptations in bacterial evolution. The capsule structures produced by these genes play an essential role in mediating cell attachment, thereby enhancing the virulence of the bacterium by stabilizing its interactions with host cells [60]. FlgI, the flagellar P-ring protein, and FlgJ, which assist in basal body rod formation, are part of the regulatory system essential for flagella biosynthesis and motility, and indirectly affect cell attachment ability through their effects on motility [61].

The pTet plasmid, first identified in *C. jejuni* 81-176, is a 45,205-bp plasmid containing *tet(O)* and T4SS [62]. The pTet family of plasmids is prevalent in *C. jejuni* and *C. coli* [63]. The pTet-family plasmids identified in this study, except the pTet_SNU15 and pTet_SNU16 plasmid types, co-harbored various AMR genes, including the tetracycline resistance gene *tet(O)* and aminoglycoside resistance genes *aadK* and *antl*. In addition, the *aphA3*-*sat4*-*aadK* AMR gene cluster, commonly found in *Campylobacter*, coexisted on the plasmid carrying T4SS, along with the *pcp* gene. Previously, we reported that *cfr(C)*, which causes resistance to five antimicrobial classes, Phenicol, Lincosamide, Oxazolidinone, Pleuromutilin, and Streptogramin A (PhLOPSA), can spread through horizontal transfer using the *pcp* and aminoglycoside resistance gene cluster as a homologous recombination site [64]. Therefore, the carriage of the pTet-type plasmid implies not only the carrying and spreading of multidrug resistance co-harbored in the plasmid, but also the formation of a physical structure through T4SS, which may pose a threat to public health. Furthermore, comparative genomic analysis of the CJ542 strain isolated in this study showed that the pTet-family plasmid fragment could be integrated into the bacterial chromosomes. The integration of a plasmid into a chromosome may increase the stability of AMR genes and T4SS, which carries the plasmid, compared to its plasmid form [65]. The integration of a plasmid fragment into the bacterial chromosome stabilizes the genes encoding AMR and VFs, preventing their loss during plasmid segregation [66]. This chromosomal integration facilitates vertical transmission, ensuring that these traits are inherited during bacterial replication [67]. In addition, pTet_SNU03 and pTet_SNU12 were transferred horizontally to the donor strain in the conjugation assay. Our results suggest that the pTet-family, the most prevalent plasmid type carrying AMR genes and T4SS, may spread via the dual pathways of vertical and horizontal transmission. This is one of the possible reasons for the global success of pTet propagation.

The pVir plasmid is a well-known plasmid in *C. jejuni*, associated with increased virulence and pathogenicity [68]. pVir includes genes encoding proteins that are linked to T4SS, showing high genetic similarity with the T4SS of the *Helicobacter pylori* P1 plasmid [69]. The P1 plasmid has been reported to be associated with competence for natural transformation and DNA uptake in *H. pylori* [70]. Bacon *et al*. (2002) reported that a mutation in the *virB11* gene, which is an essential part of the *pVir* plasmid T4SS in *C. jejuni*, led to a significant reduction in the ability of bacteria to adhere to and invade host cells [68]. In the present study, a pVir-family plasmid was identified in only one chicken-derived strain, CJ603. Further research with additional strains is needed to evaluate the potential virulence of pVir-family plasmids.

The isolates were widely distributed across different CC, with the majority belonging to CC21, CC42, and CC48. In the PubMLST database, three clone types, namely ST-21 (CC21), ST-50 (CC21), and ST-48 (CC48), were the most prevalent among 42,214 human-derived *C. jejuni* strains. cgMLST analysis of ST-21, ST-42, and ST-48 revealed significant findings, specifically within the ST-21 group. ST-21 has been reported to be one of the dominant generalists MLST variants of *C. jejuni* strains originating from all sources, implying a high risk for transmission based on its wide host range [71]. cgMLST schemes use a fixed set of conserved genome-wide genes, which involves genome-wide gene-by-gene allele calling of hundreds to thousands of conserved genes [72]. cgMLST-based high-resolution phylogenetic analysis provides reliable molecular epidemiological evidence. In this study, cgMLST-based phylogenetic analysis showed that ten ST-21 *C. jejuni* strains (one chicken-derived and nine cattle-derived strains, isolated in 2012–2013) isolated in this study showed more than 95% genetic similarity to a Korean human-derived *C. jejuni* strain isolated in 2012. This supports the idea that *C. jejuni* strains isolated from food animals can be transmitted to humans through various routes, including food chains and environmental factors, and vice versa [73]. In addition, the CJ525 strain from this study, isolated from imported chicken meat from Denmark, showed more than 95% genetic similarity to *C. jejuni* strains from UK and Denmark. Although *C. jejuni* is microaerophilic bacteria, it can survive 24–48 h under aerobic conditions [74, 75]. Furthermore, *C. jejuni* can survive 2–4 weeks under moist, reduced-oxygen conditions at 4°C, often outlasting the shelf life of the product. *C. jejuni* can also survive for 2–5 months at –20°C [76]. These results support the possibility that *C. jejuni* genotypes prevalent in Europe could be introduced into South Korea through imported meat.

Although the clonal complexes of ST-42 and ST-48 overlapped with those of other *C. jejuni* strains, few significant findings were observed. Our results suggest that traditional methods, such as MLST clone typing and PFGE, lack the precision required for detailed molecular epidemiology. Thus, WGS-based cgMLST analysis is essential to accurately assess genetic relatedness across strains from diverse sources, including those of human origin.

In conclusion, this study underscores the need for the comprehensive surveillance of *C. jejuni* in livestock to mitigate its potential zoonotic transmission to humans. By combining phenotypic and whole-genome analyses, we revealed high AMR rates and substantial prevalence of virulence factors in *C. jejuni* strains isolated from food-animals, particularly chicken-derived strains. The presence of mobile genetic elements, such as pTet and pVir plasmids, which co-carry AMR and virulence genes, suggests a high potential for the spread of resistance and pathogenicity across strains. Notably, the close genetic relationship between these strains and human *C. jejuni* isolates from public databases, coupled with evidence of European-origin strains entering Korea through imported meat, underscores the need for international and local surveillance. These findings advocate for reinforced monitoring and control strategies targeting livestock reservoirs to prevent the spread of antimicrobial-resistant virulent *C. jejuni* strains to humans.

## Supplemental Materials

Supplementary data for this paper are available on-line only at http://jmb.or.kr.



## Figures and Tables

**Table 1:** Antimicrobial resistance of *Campylobacter jejuni* strains isolated from dairy cattle and chicken.

Antimicrobial agent	Total (*n* = 94)	Cattle (*n* = 61)	Chicken (*n* = 27)	OR (95% CI)	*p*-value
Ciprofloxacin	61.4% (54/88)	61.2% (41/61)	48.1% (13/27)	0.5 (0.18–1.14)	0.093
Nalidixic acid	70.5% (62/88)	58.2% (39/61)	85.2% (23/27)	3.2 (0.99–10.59)	0.051
Tetracycline	50.0% (44/88)	38.8% (45/61)	66.7% (18/27)	2.7 (1.04–6.94)	0.041
Chloramphenicol	2.3% (2/88)	0.0% (0/61)	7.4% (2/27)	11.2 (0.52–240.76)	0.123
Gentamycin	1.1% (1/88)	0.0% (0/61)	3.7% (1/27)	7.0 (0.27–176.52)	0.239
Azithromycin	0.0% (0/88)	0.0% (0/61)	0.0% (0/27)	-	-
Erythromycin	0.0% (0/88)	0.0% (0/61)	0.0% (0/27)	-	-

Odds ratios and *p*-values were calculated using Fisher’s chi-square test. OR: Odds ratio, CI: Confidence interval.

**Table 2:** Antimicrobial resistant determinants of *C. jejuni* strains from dairy cattle and chickens.

Antimicrobial classes	Antimicrobial resistance determinants	Total (*n* = 94)	Cattle (*n* = 67)	Chicken (*n* = 27)	OR (95% CI)	*P*-value
Amino-glycoside	*ant(6)-Ia*	2.2% (2/94)	0.0% (0/67)	7.4% (2/27)	13.2 (0.61–285.23)	0.099
	*aph(2'')-Ih*	1.1% (1/94)	0.0% (0/67)	3.7% (1/27)	7.6 (0.30–193.57)	0.218
	*aph(3')-III*	4.3% (4/94)	1.5% (1/67)	11.1% (3/27)	8.3 (0.82–83.19)	0.070
	*rpsL(K43R)*	6.4% (6/94)	9.0% (6/67)	0.0% (0/27)	0.2 (0.01–3.16)	0.236
	At least one gene in AMG class	4.3% (4/94)	1.5% (1/67)	11.1% (3/27)	1.3 (0.29–5.50)	0.713
Beta-lactam	*blaOXA-61*	67.0% (63/94)	67.2% (45/67)	63.0% (17/27)	1.0 (0.38–2.53)	1.000
	*blaOXA-184*	2.2% (2/94)	0.0% (0/67)	7.4% (2/27)	13.2 (0.61–285.23)	0.099
	*blaOXA-447*	2.2% (2/94)	3.0% (2/67)	0.0% (0/27)	0.5 (0.02–10.25)	0.636
	*blaOXA-450*	5.4% (5/94)	7.5% (5/67)	0.0% (0/27)	0.2 (0.01–3.87)	0.291
	At least one gene in BETA class	76.3% (71/94)	77.6% (52/67)	70.4% (19/27)	0.8 (0.29–2.32)	0.789
Phenicol	*cat*	1.1% (1/94)	0.0% (0/67)	3.7% (1/27)	7.6 (0.30–193.57)	0.218
Tetracycline	*tet(O)*	29.8% (28/94)	28.4% (19/67)	29.6% (8/27)	1.3 (0.48–3.30)	0.628
	*tet(O/W/32/O)*	1.1% (1/94)	0.0% (0/67)	3.7% (1/27)	7.6 (0.30–193.57)	0.218
	*tet(L)*	1.1% (1/94)	0.0% (0/67)	3.7% (1/27)	7.6 (0.30–193.57)	0.218
	At least one gene in TET class	31.2% (29/94)	28.4% (19/67)	37% (10/27)	1.7 (0.68–4.42)	0.328
Quinolone	*gyrA(T86I)*	61.7% (58/94)	67.2% (45/67)	44.4% (12/27)	0.4 (0.16–0.98)	0.06

The OR was calculated using Fisher’s chi-square test. OR: Odds ratio, CI: Confidence interval, AMG: Aminoglycoside, BETA: Beta-lactam, TET: Tetracycline.

**Table 3:** Significantly differential virulence factors (VF) between cattle- and chicken-derived *C. jejuni* strains.

Virulence factor	Virulence gene	Total (*n* = 94)	Cattle (*n* = 67)	Chicken (*n* = 27)	OR (95% CI)	*P*-value
Capsule	*kpsC*	8/94 (8.5%)	1/67 (1.5%)	7/27 (25.9%)	23.1 (2.68–199.15)	0.004
Capsule biosynthesis and transport	*Cj1419c*	49/94 (52.1%)	29/67 (43.3%)	20/27 (74.1%)	3.7 (1.40–10.05)	0.009
	*Cj1420c*	62/94 (66.0%)	38/67 (56.7%)	24/27 (88.9%)	6.1 (1.67–22.27)	0.006
Cia	*ciaC*	62/94 (66.0%)	40/67 (59.7%)	22/27 (81.5%)	3.0 (1.00–8.81)	0.050
Flagella	*fliI*	69/94 (73.4%)	45/67 (67.2%)	24/27 (88.9%)	3.9 (1.06–14.41)	0.040
	*motA*	30/94 (31.9%)	16/67 (23.9%)	14/27 (51.9%)	3.4 (1.34–8.80)	0.010
	*motB*	62/94 (66.0%)	38/67 (56.7%)	24/27 (88.9%)	6.1 (1.67–22.27)	0.006
Pse	*pseB*	35/94 (37.2%)	32/67 (47.8%)	3/27 (11.1%)	0.1 (0.04–0.50)	0.003
	*pseC*	53/94 (56.4%)	47/67 (70.1%)	6/27 (22.2%)	0.1 (0.04–0.35)	< 0.001

The OR was calculated using Fisher’s chi-square test. OR: Odds ratio, CI: Confidence interval.

**Table 4:** Multi-locus sequence type (MLST) of *C. jejuni* strains from dairy cattle and chicken.

Clonal complex	Sequence type	Total (*n* = 94)	Cattle (*n* = 67)	Chicken (*n* = 27)
CC21 (39.3%, 37/94)	ST-806	17.0% (16/94)	14.9% (10/67)	3.0% (2/27)
	ST-21	12.8% (12/94)	1.5% (1/67)	0.0% (0/27)
	ST-50	6.4% (6/94)	7.5% (5/67)	1.5% (1/27)
	ST-4062	1.1% (1/94)	23.9% (16/67)	0.0% (0/27)
	ST-44	1.1% (1/94)	0.0% (0/67)	1.5% (1/27)
	ST-104	1.1% (1/94)	0.0% (0/67)	1.5% (1/27)
CC42 (21.2%, 20/94)	ST-42	17.0% (16/94)	0.0% (0/67)	1.5% (1/27)
	ST-459	3.1% (3/94)	22.4% (15/67)	1.5% (1/27)
	ST-8436	1.1% (1/94)	0.0% (0/67)	4.5% (3/27)
CC48 (8.5%, 8/94)	ST-48	8.5% (8/94)	10.4% (7/67)	1.5% (1/27)
CC353 (6.3%, 6/94)	ST-353	3.2% (3/94)	0.0% (0/67)	1.5% (1/27)
	ST-2036	1.1% (1/94)	0.0% (0/67)	4.5% (3/27)
	ST-400	1.1% (1/94)	0.0% (0/67)	1.5% (1/27)
	ST-2395	1.1% (1/94)	0.0% (0/67)	1.5% (1/27)
CC574 (4.2%, 4/94)	ST-305	4.3% (4/94)	0.0% (0/67)	6.0% (4/27)
CC283 (3.1%, 3/94)	ST-4063	3.2% (3/94)	4.5% (3/67)	0.0% (0/27)
CC22 (2.1%, 2/94)	ST-7935	2.1% (2/94)	3.0% (2/67)	0.0% (0/27)
CC581 (1.1%, 1/94)	ST-2324	1.1% (1/94)	0.0% (0/67)	1.5% (1/27)
CC443 (1.1%, 1/94)	ST-51	1.1% (1/94)	0.0% (0/67)	1.5% (1/27)
CC49 (1.1%, 1/94)	ST-467	1.1% (1/94)	0.0% (0/67)	1.5% (1/27)
CC206 (1.1%, 1/94)	ST-46	1.1% (1/94)	0.0% (0/67)	1.5% (1/27)
CC257 (1.1%, 1/94)	ST-257	1.1% (1/94)	0.0% (0/67)	1.5% (1/27)
	ST-NT	9.6% (9/94)	11.9% (8/67)	1.5% (1/27)
