# Supplementary material for: Comparative Genomic Analysis of Livestock-Derived Campylobacter jejuni: Antimicrobial Resistance, Virulence, Mobile Genetic Elements, and Genetic Relatedness
Source: J Microbiol Biotechnol. 2025 Feb 14;35:e2411044. doi: 10.4014/jmb.2411.11044 (PMC11876010; doi:10.4014/jmb.2411.11044)
Supplement: Supplementary file 1 [file jmb-35-e2411044-supple.pdf]

J. Microbiol. Biotechnol. 2025. 35: e2411044
https://doi.org/10.4014/jmb.2411.11044

Table S1. Descriptive information of 94 *C. jejuni* strains in this study.

Strain Information				Metadata			
Strain ID	Taxonomy	Multi-locus Sequence Type	Clonal Complex Type	Source	Sample type	Country	Isolated year
CJ502	*C. jejuni*	ST-21	CC-21	Chicken	Slaughterhouse	South Korea	2013
CJ503	*C. jejuni*	ST-2324	CC-581	Chicken	Imported Retail Meat	France	2004
CJ504	*C. jejuni*	ST-4062	CC-21	Cattle	Feces	South Korea	2013
CJ505	*C. jejuni*	ST-8436	CC-42	Chicken	Imported Retail Meat	South Korea	2013
CJ506	*C. jejuni*	ST-51	CC-443	Chicken	Imported Retail Meat	Brazil	2007
CJ507	*C. jejuni*	ST-48	CC-48	Cattle	Feces	South Korea	2013
CJ509	*C. jejuni*	ST-50	CC-21	Chicken	Imported Retail Meat	Belgium	missng
CJ510	*C. jejuni*	ST-806	CC-21	Cattle	Feces	South Korea	2012
CJ511	*C. jejuni*	ST-806	CC-21	Cattle	Feces	South Korea	2012
CJ512	*C. jejuni*	ST-806	CC-21	Cattle	Feces	South Korea	2012
CJ513	*C. jejuni*	ST-806	CC-21	Cattle	Feces	South Korea	2012
CJ514	*C. jejuni*	ST-4063	CC-283	Cattle	Feces	South Korea	2012
CJ515	*C. jejuni*	ST-806	CC-21	Cattle	Feces	South Korea	2013
CJ516	*C. jejuni*	ST-48	CC-48	Cattle	Feces	South Korea	2013
CJ517	*C. jejuni*	ST-50	CC-21	Cattle	Feces	South Korea	2013
CJ518	*C. jejuni*	ST-806	CC-21	Cattle	Feces	South Korea	2013
CJ519	*C. jejuni*	ST-806	CC-21	Cattle	Feces	South Korea	2013
CJ520	*C. jejuni*	ST-21	CC-21	Cattle	Feces	South Korea	2012
CJ521	*C. jejuni*	ST-2036	CC-353	Chicken	Imported Retail Meat	Brazil	2009
CJ522	*C. jejuni*	ST-42	CC-42	Cattle	Feces	South Korea	2012
CJ523	*C. jejuni*	ST-353	CC-353	Chicken	Imported Retail Meat	US	missng
CJ524	*C. jejuni*	ST-467	CC-49	Chicken	Imported Retail Meat	Brazil	2009
CJ525	*C. jejuni*	ST-21	CC-21	Chicken	Imported Retail Meat	Denmark	2009
CJ527	*C. jejuni*	ST-353	CC-353	Chicken	Imported Retail Meat	US	2008
CJ528	*C. jejuni*	ST-459	CC-42	Chicken	Imported Retail Meat	US	2006
CJ529	*C. jejuni*	ST-353	CC-353	Chicken	Imported Retail Meat	Brazil	2007
CJ530	*C. jejuni*	ST-400	CC-353	Chicken	Imported Retail Meat	Denmark	2005
CJ531	*C. jejuni*	ST-46	CC-206	Chicken	Imported Retail Meat	France	2005
CJ532	*C. jejuni*	ST-44	CC-21	Chicken	Imported Retail Meat	England	2005
CJ533	*C. jejuni*	ST-257	CC-257	Chicken	Imported Retail Meat	France	2005
CJ534	*C. jejuni*	ST-4063	CC-283	Cattle	Feces	South Korea	2012
CJ535	*C. jejuni*	ST-42	CC-42	Cattle	Feces	South Korea	2012
CJ536	*C. jejuni*	ST-42	CC-42	Cattle	Feces	South Korea	2012
CJ537	*C. jejuni*	ST-NT		Cattle	Feces	South Korea	2013
CJ538	*C. jejuni*	ST-42	CC-42	Cattle	Feces	South Korea	2013
CJ539	*C. jejuni*	ST-42	CC-42	Chicken	Imported Retail Meat	US	2006
CJ542	*C. jejuni*	ST-459	CC-42	Chicken	Imported Retail Meat	France	2004
CJ543	*C. jejuni*	ST-NT		Cattle	Feces	South Korea	2012
CJ544	*C. jejuni*	ST-42	CC-42	Cattle	Farm environment	South Korea	2012
CJ545	*C. jejuni*	ST-21	CC-21	Cattle	Feces	South Korea	2012
CJ546	*C. jejuni*	ST-7935	CC-22	Cattle	Feces	South Korea	2012
CJ547	*C. jejuni*	ST-21	CC-21	Cattle	Feces	South Korea	2012
CJ548	*C. jejuni*	ST-21	CC-21	Cattle	Feces	South Korea	2012
CJ549	*C. jejuni*	ST-21	CC-21	Cattle	Feces	South Korea	2012
CJ550	*C. jejuni*	ST-21	CC-21	Cattle	Feces	South Korea	2012
CJ551	*C. jejuni*	ST-806	CC-21	Cattle	Feces	South Korea	2012
CJ552	*C. jejuni*	ST-806	CC-21	Cattle	Feces	South Korea	2012
CJ553	*C. jejuni*	ST-48	CC-48	Cattle	Feces	South Korea	2012
CJ554	*C. jejuni*	ST-NT		Cattle	Feces	South Korea	2013

CJ555	*C. jejuni*	ST-21	CC-21	Cattle	Feces	South Korea	2013
CJ556	*C. jejuni*	ST-NT		Cattle	Feces	South Korea	2013
CJ557	*C. jejuni*	ST-NT		Cattle	Feces	South Korea	2013
CJ558	*C. jejuni*	ST-48	CC-48	Cattle	Feces	South Korea	2013
CJ559	*C. jejuni*	ST-42	CC-42	Cattle	Feces	South Korea	2013
CJ560	*C. jejuni*	ST-806	CC-21	Cattle	Feces	South Korea	2013
CJ561	*C. jejuni*	ST-48	CC-48	Cattle	Feces	South Korea	2013
CJ562	*C. jejuni*	ST-806	CC-21	Cattle	Feces	South Korea	2013
CJ563	*C. jejuni*	ST-21	CC-21	Cattle	Feces	South Korea	2013
CJ564	*C. jejuni*	ST-48	CC-48	Cattle	Feces	South Korea	2013
CJ565	*C. jejuni*	ST-50	CC-21	Cattle	Feces	South Korea	2014
CJ566	*C. jejuni*	ST-4063	CC-283	Cattle	Feces	South Korea	2014
CJ567	*C. jejuni*	ST-806	CC-21	Cattle	Feces	South Korea	2014
CJ568	*C. jejuni*	ST-42	CC-42	Cattle	Feces	South Korea	2014
CJ569	*C. jejuni*	ST-42	CC-42	Cattle	Feces	South Korea	2014
CJ570	*C. jejuni*	ST-42	CC-42	Cattle	Feces	South Korea	2014
CJ571	*C. jejuni*	ST-806	CC-21	Cattle	Feces	South Korea	2014
CJ572	*C. jejuni*	ST-50	CC-21	Cattle	Feces	South Korea	2014
CJ573	*C. jejuni*	ST-806	CC-21	Cattle	Feces	South Korea	2014
CJ574	*C. jejuni*	ST-42	CC-42	Cattle	Feces	South Korea	2014
CJ575	*C. jejuni*	ST-42	CC-42	Cattle	Feces	South Korea	2014
CJ576	*C. jejuni*	ST-806	CC-21	Cattle	Feces	South Korea	2014
CJ577	*C. jejuni*	ST-42	CC-42	Cattle	Feces	South Korea	2014
CJ578	*C. jejuni*	ST-42	CC-42	Cattle	Feces	South Korea	2014
CJ579	*C. jejuni*	ST-42	CC-42	Cattle	Feces	South Korea	2014
CJ580	*C. jejuni*	ST-50	CC-21	Cattle	Feces	South Korea	2014
CJ581	*C. jejuni*	ST-806	CC-21	Cattle	Feces	South Korea	2014
CJ582	*C. jejuni*	ST-42	CC-42	Cattle	Feces	South Korea	2015
CJ583	*C. jejuni*	ST-NT		Cattle	Feces	South Korea	2015
CJ584	*C. jejuni*	ST-NT		Cattle	Feces	South Korea	2015
CJ585	*C. jejuni*	ST-50	CC-21	Cattle	Feces	South Korea	2015
CJ586	*C. jejuni*	ST-48	CC-48	Cattle	Feces	South Korea	2016
CJ587	*C. jejuni*	ST-21	CC-21	Cattle	Feces	South Korea	2012
CJ588	*C. jejuni*	ST-NT		Cattle	Feces	South Korea	2013
CJ592	*C. jejuni*	ST-7935	CC-22	Cattle	Feces	South Korea	2012
CJ593	*C. jejuni*	ST-21	CC-21	Cattle	Feces	South Korea	2013
CJ594	*C. jejuni*	ST-48	CC-48	Chicken	Meat	South Korea	2010
CJ596	*C. jejuni*	ST-305	CC-574	Chicken	Slaughterhouse Meat	South Korea	2013
CJ597	*C. jejuni*	ST-305	CC-574	Chicken	Slaughterhouse Meat	South Korea	2013
CJ598	*C. jejuni*	ST-305	CC-574	Chicken	Slaughterhouse Meat	South Korea	2013
CJ601	*C. jejuni*	ST-305	CC-574	Chicken	Slaughterhouse Meat	South Korea	2013
CJ603	*C. jejuni*	ST-2395	CC-353	Chicken	Imported Retail Meat	Brazil	2008
CJ604	*C. jejuni*	ST-459	CC-42	Chicken	Imported Retail Meat	Brazil	2007
CJ605	*C. jejuni*	ST-NT		Chicken	Imported Retail Meat	England	2005
CJ606	*C. jejuni*	ST-104	CC-21	Chicken	Imported Retail Meat	France	2005

Table S2. Correlation between phenotypic antimicrobial resistance and resistance determinants of *C. jejuni* strains in this study.

Antimicrobial classes	Antimicrobial resistance determinants	Correlation coefficient	*P*-value
Aminoglycoside (Gentamicin)	*ant(6)-Ia*	0.70	<0.001
	aph(2'')-Ih	1.00	<0.001
	aph(3')-III	0.49	<0.001
	rpsL(K43R)	0.03	0.789
	At least one gene within class	0.49	<0.001
Tetracycline	*tet(O)*	0.62	<0.001
	tet(O/W/32/O)	0.11	0.314
	tet(L)	0.11	0.314
	At least one gene within class	0.66	<0.001
Quinolone (Ciprofloxacin)	*gyrA(T86I)*	0.93	<0.001
Quinolone (Nalidixic acid)	*gyrA(T86I)*	0.63	<0.001
Chloramphenicol	*cat*	1.00	<0.001

Table S3. Prevalence of virulence factor classes and genes in chicken and cattle-derived *C. jejuni*.

Virulence factor classes	Virulence factor genes	Cattle	Chicken	Total
CadF	*cadF*	100% (67/67)	100% (27/27)	100% (94/94)
CadF	At least one gene within class	100% (67/67)	100% (27/27)	100% (94/94)
Capsule	*Cj1416c*	98.5% (66/67)	96.3% (26/27)	97.9% (92/94)
Capsule	*Cj1421c*	13.4% (9/67)	7.4% (2/27)	11.7% (11/94)
Capsule	*Cj1422c*	7.5% (5/67)	7.4% (2/27)	7.4% (7/94)
Capsule	*Cj1426c*	14.9% (10/67)	7.4% (2/27)	12.8% (12/94)
Capsule	*Cj1427c*	14.9% (10/67)	7.4% (2/27)	12.8% (12/94)
Capsule	*Cj1432c*	14.9% (10/67)	7.4% (2/27)	12.8% (12/94)
Capsule	*Cj1434c*	10.4% (7/67)	3.7% (1/27)	8.5% (8/94)
Capsule	*Cj1435c*	14.9% (10/67)	7.4% (2/27)	12.8% (12/94)
Capsule	*Cj1436c*	14.9% (10/67)	7.4% (2/27)	12.8% (12/94)
Capsule	*Cj1437c*	14.9% (10/67)	7.4% (2/27)	12.8% (12/94)
Capsule	*Cj1438c*	10.4% (7/67)	3.7% (1/27)	8.5% (8/94)
Capsule	*Cj1440c*	14.9% (10/67)	7.4% (2/27)	12.8% (12/94)
Capsule	*cysC*	94% (63/67)	81.5% (22/27)	90.4% (85/94)
Capsule	*fcl*	14.9% (10/67)	7.4% (2/27)	12.8% (12/94)
Capsule	*glf*	14.9% (10/67)	7.4% (2/27)	12.8% (12/94)
Capsule	*gmhA2*	14.9% (10/67)	7.4% (2/27)	12.8% (12/94)
Capsule	*hddA*	14.9% (10/67)	7.4% (2/27)	12.8% (12/94)
Capsule	*hddC*	16.4% (11/67)	7.4% (2/27)	13.8% (13/94)
Capsule	*kfiD*	14.9% (10/67)	7.4% (2/27)	12.8% (12/94)
Capsule	*kpsC*	1.5% (1/67)	25.9% (7/27)	8.5% (8/94)
Capsule	*kpsD*	89.6% (60/67)	85.2% (23/27)	88.3% (83/94)
Capsule	*kpsE*	14.9% (10/67)	14.8% (4/27)	14.9% (14/94)
Capsule	*kpsF*	14.9% (10/67)	7.4% (2/27)	12.8% (12/94)
Capsule	*kpsM*	14.9% (10/67)	7.4% (2/27)	12.8% (12/94)
Capsule	*kpsS*	67.2% (45/67)	77.8% (21/27)	70.2% (66/94)
Capsule	*kpsT*	14.9% (10/67)	7.4% (2/27)	12.8% (12/94)
Capsule	*rfbC*	14.9% (10/67)	7.4% (2/27)	12.8% (12/94)
Capsule	At least one gene within class	100% (67/67)	100% (27/27)	100% (94/94)
Capsule biosynthesis and transport	*Cj1417c*	94% (63/67)	92.6% (25/27)	93.6% (88/94)
Capsule biosynthesis and transport	*Cj1419c*	43.3% (29/67)	74.1% (20/27)	52.1% (49/94)
Capsule biosynthesis and transport	*Cj1420c*	56.7% (38/67)	88.9% (24/27)	66% (62/94)
Capsule biosynthesis and transport	At least one gene within class	95.5% (64/67)	92.6% (25/27)	94.7% (89/94)
CDT	*cdtA*	97% (65/67)	100% (27/27)	97.9% (92/94)
CDT	*cdtB*	97% (65/67)	100% (27/27)	97.9% (92/94)
CDT	*cdtC*	97% (65/67)	100% (27/27)	97.9% (92/94)
CDT	At least one gene within class	97% (65/67)	100% (27/27)	97.9% (92/94)
CiaB	*ciaB*	100% (67/67)	100% (27/27)	100% (94/94)
CiaC	*ciaC*	59.7% (40/67)	81.5% (22/27)	66% (62/94)
Cia	At least one gene within class	100% (67/67)	100% (27/27)	100% (94/94)
Flagella	*flaA*	3% (2/67)	0% (0/27)	2.1% (2/94)
Flagella	*flaB*	0% (0/67)	3.7% (1/27)	1.1% (1/94)
Flagella	*flaC*	98.5% (66/67)	100% (27/27)	98.9% (93/94)
Flagella	*flaD*	88.1% (59/67)	74.1% (20/27)	84% (79/94)
Flagella	*flaG*	83.6% (56/67)	85.2% (23/27)	84% (79/94)
Flagella	*flgB*	56.7% (38/67)	44.4% (12/27)	53.2% (50/94)
Flagella	*flgC*	100% (67/67)	100% (27/27)	100% (94/94)
Flagella	*flgD*	97% (65/67)	100% (27/27)	97.9% (92/94)
Flagella	*flgE*	97% (65/67)	81.5% (22/27)	92.6% (87/94)
Flagella	*flgF*	100% (67/67)	100% (27/27)	100% (94/94)
Flagella	*flgG*	100% (67/67)	100% (27/27)	100% (94/94)
Flagella	*flgH*	100% (67/67)	96.3% (26/27)	98.9% (93/94)
Flagella	*flgI*	94% (63/67)	96.3% (26/27)	94.7% (89/94)
Flagella	*flgK*	94% (63/67)	88.9% (24/27)	92.6% (87/94)
Flagella	*flhA*	98.5% (66/67)	100% (27/27)	98.9% (93/94)
Flagella	*flhB*	100% (67/67)	96.3% (26/27)	98.9% (93/94)
Flagella	*flhF*	100% (67/67)	100% (27/27)	100% (94/94)
Flagella	*flhG*	100% (67/67)	100% (27/27)	100% (94/94)
Flagella	*fliA*	98.5% (66/67)	100% (27/27)	98.9% (93/94)
Flagella	*fliD*	59.7% (40/67)	40.7% (11/27)	54.3% (51/94)
Flagella	*fliE*	100% (67/67)	100% (27/27)	100% (94/94)
Flagella	*fliF*	95.5% (64/67)	88.9% (24/27)	93.6% (88/94)
Flagella	*fliG*	100% (67/67)	100% (27/27)	100% (94/94)
Flagella	*fliH*	95.5% (64/67)	100% (27/27)	96.8% (91/94)
Flagella	*fliI*	67.2% (45/67)	88.9% (24/27)	73.4% (69/94)
Flagella	*fliL*	76.1% (51/67)	77.8% (21/27)	76.6% (72/94)
Flagella	*fliM*	97% (65/67)	100% (27/27)	97.9% (92/94)
Flagella	*fliN*	100% (67/67)	100% (27/27)	100% (94/94)
Flagella	*fliP*	100% (67/67)	100% (27/27)	100% (94/94)
Flagella	*fliQ*	97% (65/67)	100% (27/27)	97.9% (92/94)
Flagella	*fliR*	100% (67/67)	100% (27/27)	100% (94/94)
Flagella	*fliS*	97% (65/67)	96.3% (26/27)	96.8% (91/94)
Flagella	*fliY*	94% (63/67)	100% (27/27)	95.7% (90/94)
Flagella	*motA*	23.9% (16/67)	51.9% (14/27)	31.9% (30/94)
Flagella	*motB*	56.7% (38/67)	88.9% (24/27)	66% (62/94)
Flagella	*pflA*	97% (65/67)	100% (27/27)	97.9% (92/94)
Flagella	*pseG*	79.1% (53/67)	92.6% (25/27)	83% (78/94)
Flagella	*pseH*	9% (6/67)	0% (0/27)	6.4% (6/94)
Flagella	*ptmA*	16.4% (11/67)	29.6% (8/27)	20.2% (19/94)
Flagella	*ptmB*	14.9% (10/67)	51.9% (14/27)	25.5% (24/94)
Flagella	At least one gene within class	100% (67/67)	100% (27/27)	100% (94/94)
JlpA	*jlpA*	95.5% (64/67)	92.6% (25/27)	94.7% (89/94)
JlpA	At least one gene within class	95.5% (64/67)	92.6% (25/27)	94.7% (89/94)
LOS	*Cj1135*	9% (6/67)	18.5% (5/27)	11.7% (11/94)
LOS	*Cj1136*	9% (6/67)	18.5% (5/27)	11.7% (11/94)

LOS	*Cj1137c*	9% (6/67)	18.5% (5/27)	11.7% (11/94)
LOS	*Cj1138*	9% (6/67)	18.5% (5/27)	11.7% (11/94)
LOS	*cstIII*	9% (6/67)	18.5% (5/27)	11.7% (11/94)
LOS	*gmhA*	9% (6/67)	18.5% (5/27)	11.7% (11/94)
LOS	*gmhB*	73.1% (49/67)	48.1% (13/27)	66% (62/94)
LOS	*hldD*	26.9% (18/67)	29.6% (8/27)	27.7% (26/94)
LOS	*hldE*	26.9% (18/67)	7.4% (2/27)	21.3% (20/94)
LOS	*htrB*	20.9% (14/67)	40.7% (11/27)	26.6% (25/94)
LOS	*neuA1*	9% (6/67)	18.5% (5/27)	11.7% (11/94)
LOS	*neuB1*	9% (6/67)	18.5% (5/27)	11.7% (11/94)
LOS	*neuC1*	9% (6/67)	18.5% (5/27)	11.7% (11/94)
LOS	*waaC*	31.3% (21/67)	51.9% (14/27)	37.2% (35/94)
LOS	*waaF*	9% (6/67)	18.5% (5/27)	11.7% (11/94)
LOS	*waaV*	9% (6/67)	18.5% (5/27)	11.7% (11/94)
LOS	*wlaN*	9% (6/67)	18.5% (5/27)	11.7% (11/94)
LOS	At least one gene within class	97% (65/67)	92.6% (25/27)	95.7% (90/94)
MOMP	*porA*	10.4% (7/67)	11.1% (3/27)	10.6% (10/94)
MOMP	At least one gene within class	10.4% (7/67)	11.1% (3/27)	10.6% (10/94)
PEB1	*pebA*	97% (65/67)	100% (27/27)	97.9% (92/94)
PEB1	At least one gene within class	97% (65/67)	100% (27/27)	97.9% (92/94)
Pse	*cheA*	76.1% (51/67)	92.6% (25/27)	80.9% (76/94)
Pse	*cheV*	100% (67/67)	100% (27/27)	100% (94/94)
Pse	*cheW*	98.5% (66/67)	100% (27/27)	98.9% (93/94)
Pse	*cheY*	100% (67/67)	100% (27/27)	100% (94/94)
Pse	*eptC*	80.6% (54/67)	51.9% (14/27)	72.3% (68/94)
Pse	*flgA*	100% (67/67)	100% (27/27)	100% (94/94)
Pse	*flgJ*	64.2% (43/67)	59.3% (16/27)	62.8% (59/94)
Pse	*flgM*	86.6% (58/67)	92.6% (25/27)	88.3% (83/94)
Pse	*flgP*	100% (67/67)	96.3% (26/27)	98.9% (93/94)
Pse	*flgQ*	97% (65/67)	100% (27/27)	97.9% (92/94)
Pse	*flgR*	100% (67/67)	100% (27/27)	100% (94/94)
Pse	*flgS*	100% (67/67)	100% (27/27)	100% (94/94)
Pse	*fliK*	88.1% (59/67)	77.8% (21/27)	85.1% (80/94)
Pse	*fliW*	100% (67/67)	100% (27/27)	100% (94/94)
Pse	*maf4*	11.9% (8/67)	0% (0/27)	8.5% (8/94)
Pse	*pseA*	67.2% (45/67)	92.6% (25/27)	74.5% (70/94)
Pse	*pseB*	47.8% (32/67)	11.1% (3/27)	37.2% (35/94)
Pse	*pseC*	70.1% (47/67)	22.2% (6/27)	56.4% (53/94)
Pse	*pseD/maf2*	3% (2/67)	7.4% (2/27)	4.3% (4/94)
Pse	*pseE/maf5*	29.9% (20/67)	11.1% (3/27)	24.5% (23/94)
Pse	*pseF*	56.7% (38/67)	59.3% (16/27)	57.4% (54/94)
Pse	*pseI*	0% (0/67)	3.7% (1/27)	1.1% (1/94)
Pse	*rpoN*	98.5% (66/67)	100% (27/27)	98.9% (93/94)
Pse	At least one gene within class	100% (67/67)	100% (27/27)	100% (94/94)

Table S4. Information on the genetic subtypes of pTet and pVir plasmids identified in the *C. jejuni* strains from this study.

Species	Plasmid size (bp)	Plasmid type	Plasmid subtype	Number of isolates	AMR gene on plasmid	VF genes on plasmid	Isolates (MLST ST type of bacterial strain)									
C. jejuni	55,637	pTet	pTet_SNU01	10	*tet(O)*	*vapD*, T4SS	pCJ524 (ST-467)	pCJ568 (ST-42)	pCJ574 (ST-42)	pCJ577 (ST-42)	pCJ578 (ST-42)	pCJ579 (ST-42)	pCJ582 (ST-42)	pCJ569 (ST-42)	pCJ570 (ST-42)	pCJ575 (ST-42)
	45,150	pTet	pTet_SNU02	9	*tet(O)*	*vapD*, T4SS	pCJ502 (ST-21)	pCJ520 (ST-21)	pCJ545 (ST-21)	pCJ547 (ST-21)	pCJ563 (ST-21)	pCJ587 (ST-21)	pCJ593 (ST-21)	pCJ548 (ST-21)	pCJ549 (ST-21)	
	43,549	pTet	pTet_SNU03	4	*tet(O)*	*vapD*, T4SS	pCJ605 (ST-NT)	pCJ597 (ST-305)	pCJ598 (ST-305)	pCJ601 (ST-305)						
	44,717	pTet	pTet_SNU04	2	*tet(O)*	*vapD*, T4SS	pCJ550 (ST-21)	pCJ592 (ST-7935)								
	44,096	pTet	pTet_SNU05	1	*tet(O)*	*vapD*, T4SS	pCJ588 (ST-NT)									
	45,195	pTet	pTet_SNU06	1	*tet(O)*	*vapD*, T4SS	pCJ555 (ST-21)									
	45,196	pTet	pTet_SNU07	1	*tet(O)*	*vapD*, T4SS	pCJ521 (ST-2036)									
	47,473	pTet	pTet_SNU08	1	*tet(O)*	*vapD*, T4SS	pCJ546 (ST-7935)									
	47,521	pTet	pTet_SNU09	1	*tet(O)*	*vapD*, T4SS	pCJ533 (ST-257)									
	50,383	pTet	pTet_SNU10	1	*tet(O)*	*vapD*, T4SS	pCJ553 (ST-48)									
	45,767	pTet	pTet_SNU11	1	*tet(O)*	*vapD*, T4SS	pCJ603_1 (ST-2395)									
	47,360	pTet	pTet_SNU12	5	*tet(O)*	*vapD*, T4SS	pCJ596 (ST-305)	pCJ554 (ST-NT)	pCJ543 (ST-NT)	pCJ557 (ST-NT)	pCJ556 (ST-NT)					
	48,652	pTet	pTet_SNU13	4	*tet(O)*	*vapD*, T4SS	pCJ558 (ST-48)	pCJ561 (ST-48)	pCJ564 (ST-48)	pCJ594 (ST-48)						
	63,353	pTet	pTet_SNU14	1	*ant(6)-Ia, aph(3')-III, aph(2'')-Ih, tet(O)*	*vapD*, T4SS	pCJ509 (ST-50)									
	39,591	pTet	pTet_SNU15	1	-	*vapD*, T4SS	pCJ525 (ST-21)									
	44,994	pTet	pTet_SNU16	1	-	*vapD*, T4SS	pCJ606 (ST-104)									
	46,524	pTet	pTet_SNU17	1	*aph(3')-III, tet(O)*	*vapD*, T4SS	pCJ515 (ST-806)									
	37,042	pVir	pVir_SNU01	1	-	T4SS	pCJ603_2 (ST-2395)									

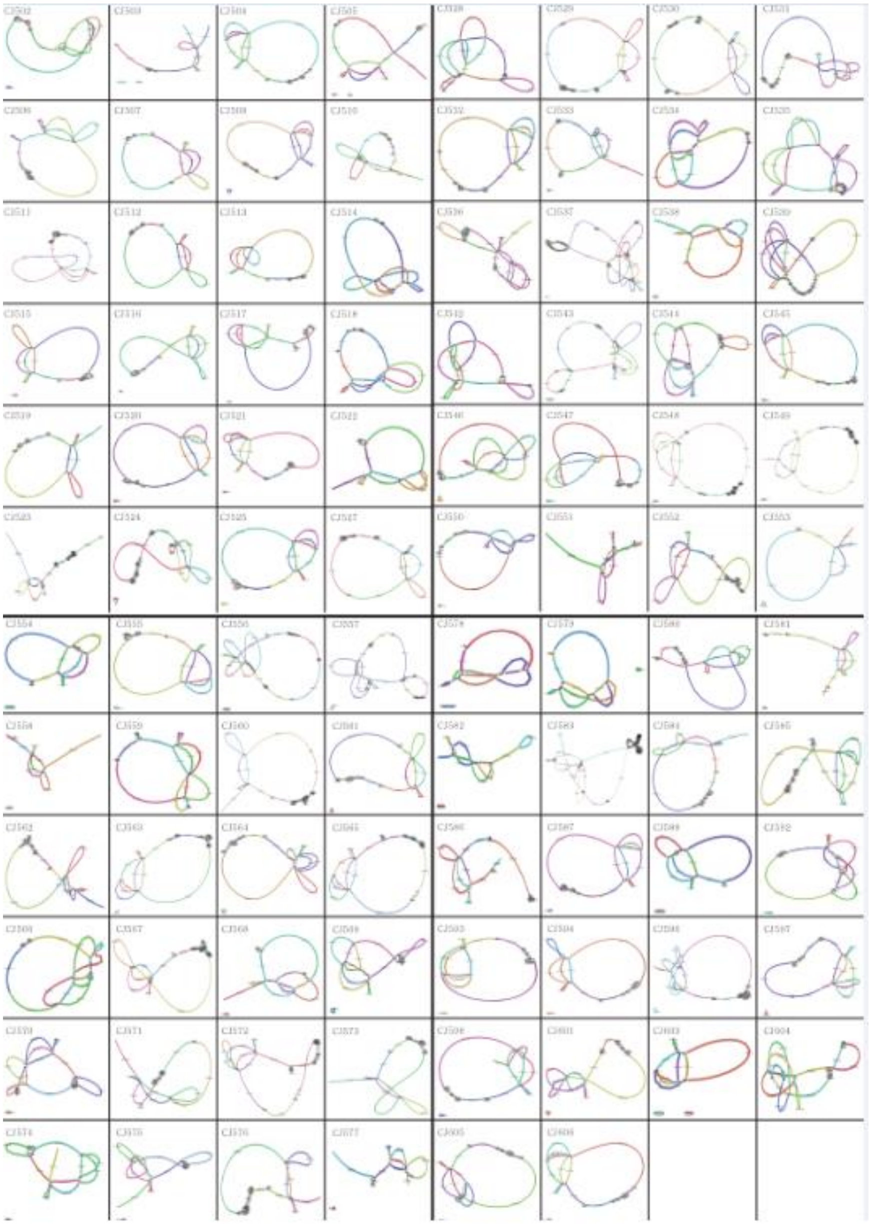

Fig. S1. De Bruijn graph of each 94 *C. jejuni* strains in this study by Bandage program.

ST-21 phylogenetic clade I

ST-21 phylogenetic clade II

ST-21 phylogenetic clade III

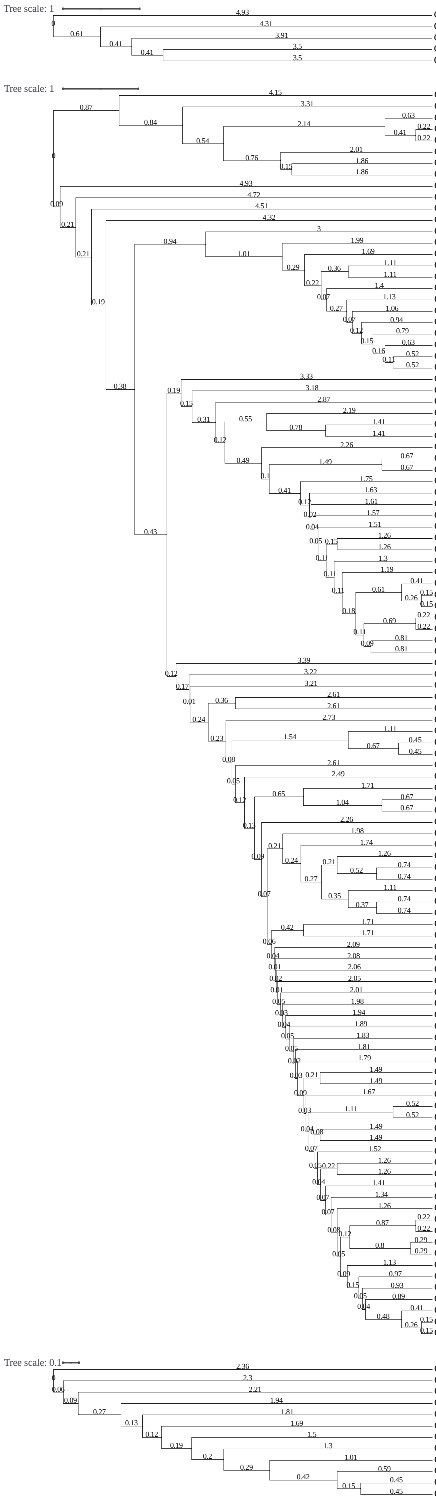

ST-21 phylogenetic clade I

Strain	Country	Host	Year	Data source
GCA 005214785.1	USA	Cattle	2017	NCBI
GCA 022398905.1	USA	Cattle	2022	NCBI
CJ550	Korea	Cattle	2012	This study
GCA 033041535.1	Chile	Human	2020	NCBI
GCA 033041495.1	Chile	Human	2021	NCBI

ST-21 phylogenetic clade II

Strain	Country	Host	Year	Data source
GCA 006347695.1	Ireland	Human	2012	NCBI
GCA 010352885.1	UK	Human	2016	NCBI
GCA 001233745.1	UK	Human	2011	NCBI
GCA 001237565.1	UK	Human	2011	NCBI
GCA 001223745.1	UK	Human	2011	NCBI
GCA 033402335.1	Ireland	Human	2019	NCBI
GCA 033405715.1	Ireland	Human	2019	NCBI
GCA 015140895.1	UK	Human	2015	NCBI
GCA 008283335.1	UK	Human	2016	NCBI
GCA 008237445.1	UK	Human	2017	NCBI
GCA 033573375.1	Switzerland	Human	2013	NCBI
CJ525	Korea	Chicken (imported from Denmark)	2009	This study
GCA 001229025.1	UK	Human	2011	NCBI
GCA 010303125.1	UK	Human	2017	NCBI
GCA 008207105.1	UK	Human	2018	NCBI
GCA 033185215.1	Spain	Pheasant	2008	NCBI
GCA 008335105.1	UK	Human	2015	NCBI
GCA 008143605.1	UK	Human	2016	NCBI
GCA 033218075.1	Denmark	Human	2019	NCBI
GCA 010325705.1	UK	Human	2016	NCBI
GCA 033221315.1	Denmark	Human	2019	NCBI
GCA 033290005.1	Denmark	Human	2017	NCBI
GCA 008210645.1	UK	Human	2018	NCBI
GCA 010995815.1	France	Human	2016	NCBI
GCA 008337805.1	UK	Human	2016	NCBI
GCA 010325475.1	UK	Human	2017	NCBI
GCA 033222265.1	Denmark	Human	2019	NCBI
GCA 010336605.1	UK	Human	2017	NCBI
GCA 008235185.1	UK	Human	2017	NCBI
GCA 033406055.1	Ireland	Human	2019	NCBI
GCA 008239125.1	UK	Human	2017	NCBI
GCA 007984415.1	UK	Human	2018	NCBI
GCA 008238125.1	UK	Human	2017	NCBI
GCA 008235125.1	UK	Human	2017	NCBI
GCA 008278465.1	UK	Human	2016	NCBI
GCA 033321005.1	Denmark	Turkey	2016	NCBI
GCA 033224905.1	Denmark	Human	2019	NCBI
GCA 007967485.1	UK	Human	2017	NCBI
GCA 033403815.1	Ireland	Human	2019	NCBI
GCA 010292065.1	UK	Human	2017	NCBI
GCA 008336065.1	UK	Human	2016	NCBI
GCA 001238885.1	UK	Human	2011	NCBI
GCA 001231425.1	UK	Human	2011	NCBI
GCA 033406635.1	Ireland	Human	2019	NCBI
GCA 032835655.1	Ireland	Wild bird	2018	NCBI
GCA 032835635.1	Ireland	Wild bird	2018	NCBI
GCA 001228665.1	UK	Human	2011	NCBI
GCA 001228605.1	UK	Human	2011	NCBI
GCA 033406355.1	Ireland	Human	2019	NCBI
GCA 010333875.1	UK	Human	2017	NCBI
GCA 010214155.1	UK	Human	2016	NCBI
GCA 018516985.1	Austria	Cattle	2010	NCBI
GCA 007946145.1	UK	Human	2018	NCBI
GCA 008219225.1	UK	Human	2018	NCBI
GCA 008142505.1	UK	Human	2016	NCBI
GCA 033289165.1	Denmark	Human	2017	NCBI
GCA 033291105.1	Denmark	Chicken	2017	NCBI
GCA 033296125.1	Denmark	Human	2016	NCBI
GCA 033295645.1	Denmark	Human	2016	NCBI
GCA 033211755.1	USA	Human	2017	NCBI
GCA 010302645.1	UK	Human	2017	NCBI
GCA 008235905.1	UK	Human	2017	NCBI
GCA 008277065.1	UK	Human	2016	NCBI
GCA 008138385.1	UK	Human	2016	NCBI
GCA 033620085.1	Italy	Chicken	2010	NCBI
GCA 023984075.1	USA	Chicken	2022	NCBI
GCA 022883055.1	USA	Sheep	2022	NCBI
GCA 008143525.1	UK	Human	2016	NCBI
GCA 008239305.1	UK	Human	2017	NCBI
GCA 008231725.1	UK	Human	2017	NCBI
GCA 022398885.1	USA	Cattle	2022	NCBI
GCA 027255615.1	USA	Chicken	2022	NCBI
GCA 023172135.1	USA	Cattle	2022	NCBI
GCA 008341345.1	UK	Human	2015	NCBI
GCA 007933485.1	UK	Human	2018	NCBI
GCA 033404335.1	Ireland	Human	2019	NCBI
GCA 010329145.1	UK	Human	2017	NCBI
GCA 033233925.1	Denmark	Human	2019	NCBI
GCA 033001945.1	Luxembourg	Chicken	2014	NCBI
GCA 010296345.1	UK	Human	2017	NCBI
GCA 010332065.1	UK	Human	2017	NCBI
GCA 008347665.1	UK	Human	2016	NCBI
GCA 032826795.1	Denmark	Human	2014	NCBI
GCA 010338825.1	UK	Human	2017	NCBI
GCA 007900045.1	UK	Human	2019	NCBI
GCA 032835615.1	Ireland	Wild bird	2018	NCBI
GCA 033569595.1	Switzerland	Human	2006	NCBI
GCA 033307295.1	Denmark	Cattle	2016	NCBI
GCA 032835595.1	Ireland	Wild bird	2018	NCBI
GCA 011721935.1	UK	Human	2018	NCBI
GCA 007957185.1	UK	Human	2018	NCBI
GCA 001229325.1	UK	Human	2011	NCBI
GCA 001224865.1	UK	Human	2011	NCBI
GCA 010344205.1	UK	Human	2017	NCBI
GCA 033403155.1	Ireland	Human	2019	NCBI
GCA 033007395.1	Italy	Chicken	2014	NCBI
GCA 032754915.1	Spain	Cattle	2014	NCBI
GCA 010998235.1	France	Human	2012	NCBI
GCA 904845255.1	Switzerland	Cattle	2017	NCBI
GCA 033405355.1	Ireland	Human	2019	NCBI
GCA 033404775.1	Ireland	Human	2019	NCBI
GCA 032834675.1	Ireland	Wild bird	2017	NCBI
GCA 032834635.1	Ireland	Wild bird	2017	NCBI
GCA 033402575.1	Ireland	Human	2019	NCBI
GCA 007933165.1	UK	Human	2018	NCBI
GCA 033404755.1	Ireland	Human	2019	NCBI
GCA 033403475.1	Ireland	Human	2019	NCBI
GCA 001238245.1	UK	Human	2011	NCBI
GCA 001234225.1	UK	Human	2011	NCBI
GCA 001233965.1	UK	Human	2011	NCBI

ST-21 phylogenetic clade III

Strain	Country	Host	Year	Data source
GCA 004845505.1	USA	Human	2014	NCBI
CJ549	Korea	Cattle	2012	This study
CJ502	Korea	Chicken	2013	This study
CJ545	Korea	Cattle	2012	This study
CJ520	Korea	Cattle	2012	This study
CJ555	Korea	Cattle	2013	This study
CJ593	Korea	Cattle	2013	This study
GCA 032735145.1	Korea	Human	2012	NCBI
CJ548	Korea	Cattle	2012	This study
CJ563	Korea	Cattle	2013	This study
CJ587	Korea	Cattle	2012	This study
CJ547	Korea	Cattle	2012	This study

Fig. S2. UPGMA dendrogram of ST-21 *C. jejuni* strains from this study and NCBI database.

UPGMA dendrogram was constructed based on cgMLST 1,342 allelic profile as categorical values by using BioNumerics (v.6.6). The phylogenetic clades were clustered based on a 95% similarity criterion.

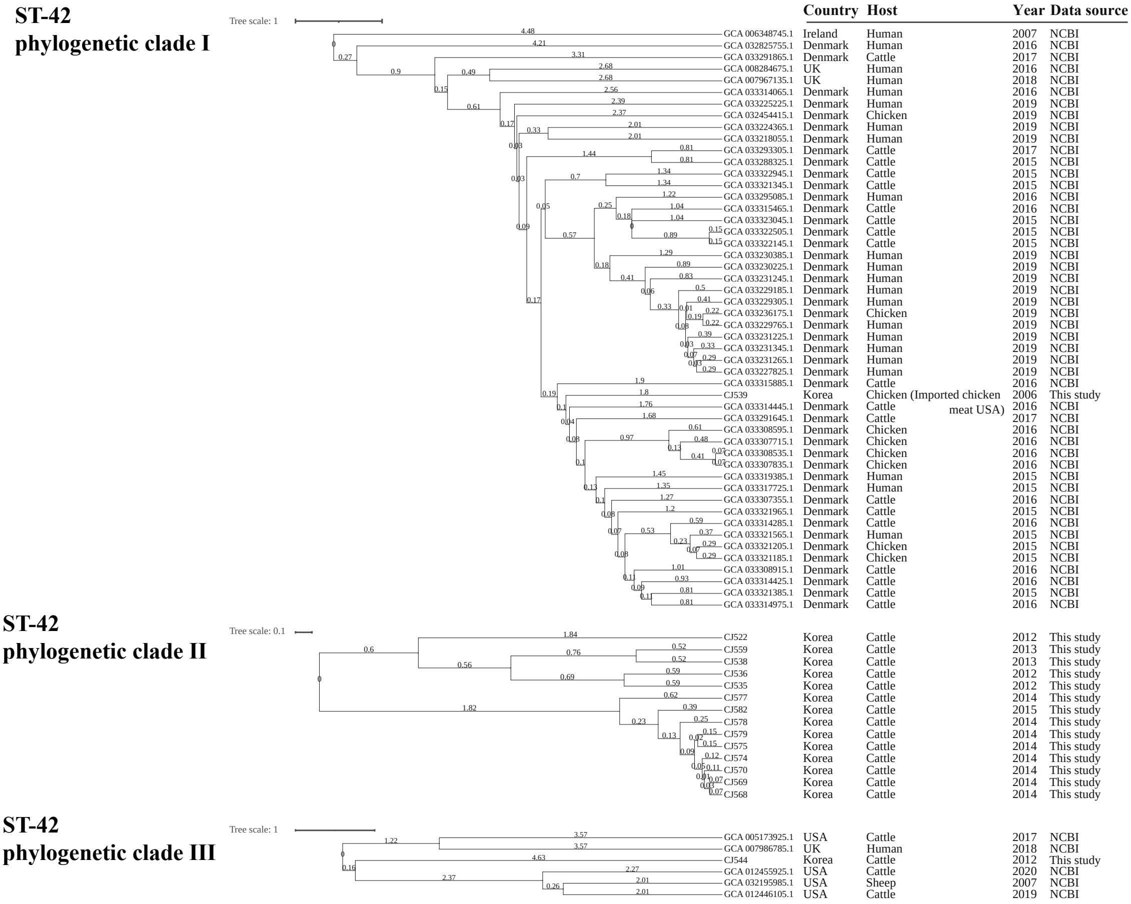

Fig. S3. UPGMA dendrogram of ST-42 *C. jejuni* strains from this study and NCBI database.

UPGMA dendrogram was constructed based on cgMLST 1,342 allelic profile as categorical values by using BioNumerics (v.6.6). The phylogenetic clades were clustered based on a 95% similarity criterion.

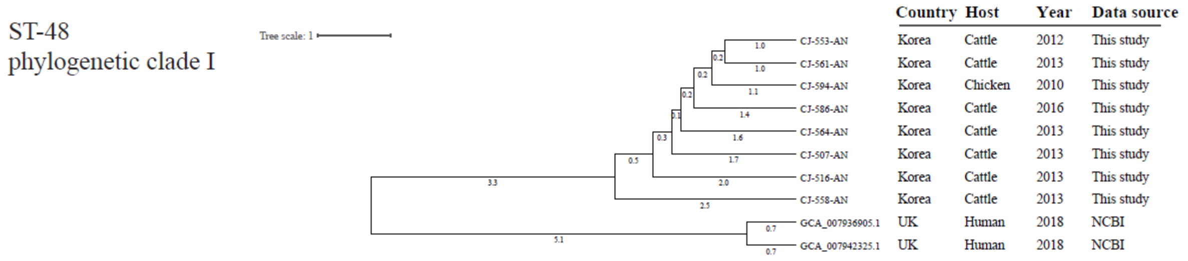

Fig. S4. UPGMA dendrogram of ST-48 *C. jejuni* strains from this study and NCBI database.

UPGMA dendrogram was constructed based on cgMLST 1,342 allelic profile as categorical values by using BioNumerics (v.6.6). The phylogenetic clades were clustered based on a 95% similarity criterion.

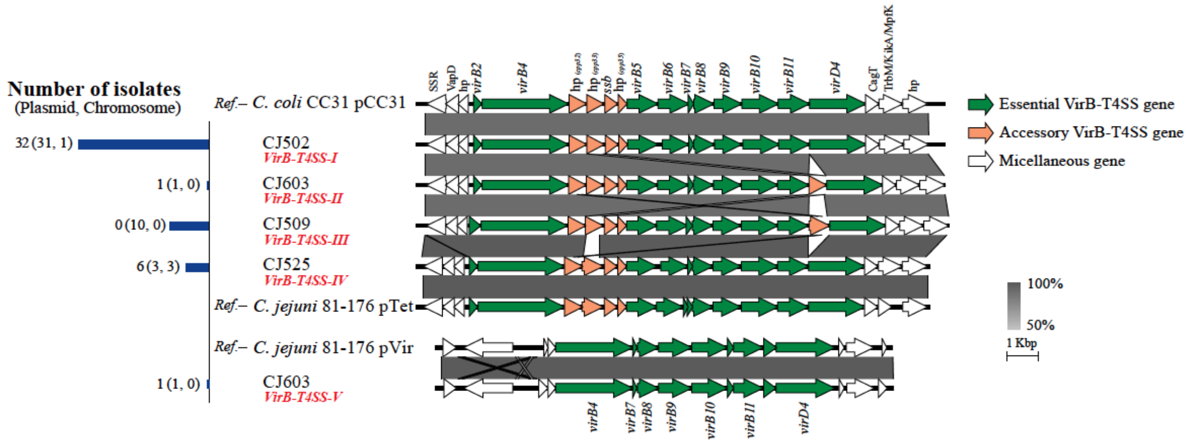

Fig. S5. Comparative genomic analysis of Type IV secretion system gene cassettes
The nucleotide sequences were aligned and compared using easyfig (v2.2.3).

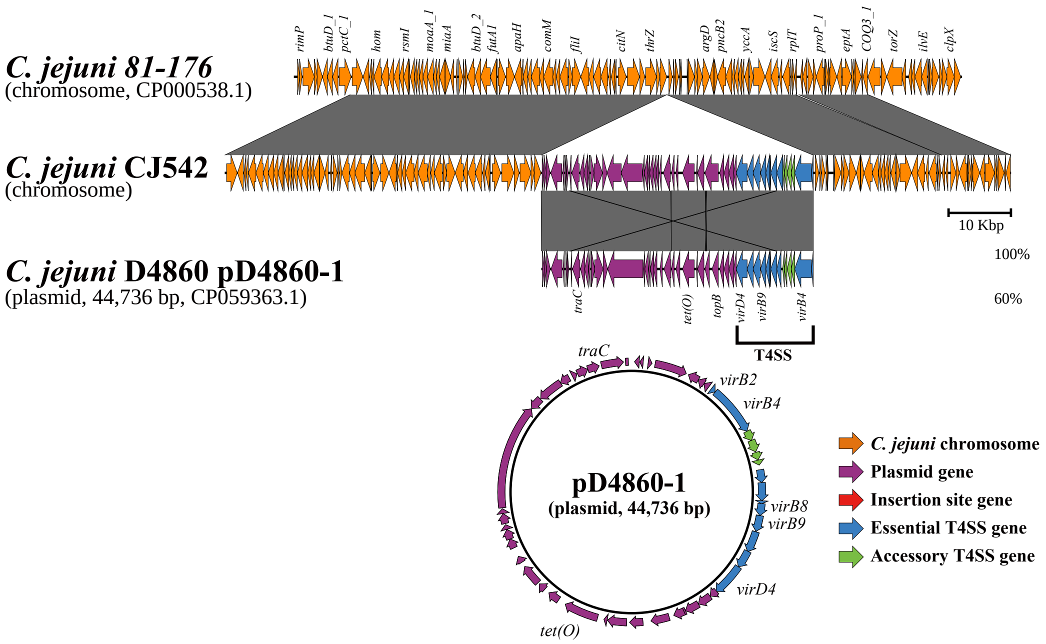

Fig. S6. Chromosomal insertion of T4SS carrying pTet plasmid.
Plasmid integration into the chromosome of CJ542 strains. The nucleotide sequences were aligned and compared using easyfig (v2.2.3).
